# *KLK3* SNP–SNP interactions for prediction of prostate cancer aggressiveness

**DOI:** 10.1038/s41598-021-85169-7

**Published:** 2021-04-29

**Authors:** Hui-Yi Lin, Po-Yu Huang, Chia-Ho Cheng, Heng-Yuan Tung, Zhide Fang, Anders E. Berglund, Ann Chen, Jennifer French-Kwawu, Darian Harris, Julio Pow-Sang, Kosj Yamoah, John L. Cleveland, Shivanshu Awasthi, Robert J. Rounbehler, Travis Gerke, Jasreman Dhillon, Rosalind Eeles, Zsofia Kote-Jarai, Kenneth Muir, UKGPCS collaborators, Rosalind Eeles, Zsofia Kote-Jarai, Kenneth Muir, Johanna Schleutker, Nora Pashayan, APCB (Australian Prostate Cancer BioResource), Judith Clements, Jyotsna Batra, David E. Neal, Sune F. Nielsen, Børge G. Nordestgaard, Henrik Gronberg, Fredrik Wiklund, Graham G. Giles, Christopher A. Haiman, Ruth C. Travis, Janet L. Stanford, Adam S. Kibel, Cezary Cybulski, Kay-Tee Khaw, Christiane Maier, Stephen N. Thibodeau, Manuel R. Teixeira, Lisa Cannon-Albright, Hermann Brenner, Radka Kaneva, Hardev Pandha, The PRACTICAL consortium, Hui-Yi Lin, Rosalind Eeles, Zsofia Kote-Jarai, Kenneth Muir, Johanna Schleutker, Nora Pashayan, David E. Neal, Sune F. Nielsen, Børge G. Nordestgaard, Henrik Gronberg, Fredrik Wiklund, Graham G. Giles, Christopher A. Haiman, Ruth C. Travis, Janet L. Stanford, Adam S. Kibel, Cezary Cybulski, Kay-Tee Khaw, Christiane Maier, Stephen N. Thibodeau, Manuel R. Teixeira, Lisa Cannon-Albright, Hermann Brenner, Radka Kaneva, Hardev Pandha, Srilakshmi Srinivasan, Judith Clements, Jyotsna Batra, Jong Y. Park

**Affiliations:** 1 Biostatistics Program, School of Public Health, Louisiana State University Health Sciences Center, New Orleans, LA 70112 USA; 2 Computational Intelligence Technology Center, Industrial Technology Research Institute, Hsinchu, Taiwan; 3 Department of Biostatistics and Bioinformatics, Moffitt Cancer Center & Research Institute, Tampa, FL 33612 USA; 4 Department of Genitourinary Oncology, Moffitt Cancer Center & Research Institute, Tampa, FL 33612 USA; 5 Department of Radiation Oncology, Moffitt Cancer Center & Research Institute, Tampa, FL 33612 USA; 6 Department of Tumor Biology, Moffitt Cancer Center & Research Institute, Tampa, FL 33612 USA; 7 Department of Cancer Epidemiology, Moffitt Cancer Center & Research Institute, Tampa, FL 33612 USA; 8 Department of Pathology, Moffitt Cancer Center & Research Institute, Tampa, FL 33612 USA; 9 The Institute of Cancer Research, London, SM2 5NG UK; 10 Royal Marsden NHS Foundation Trust, London, SW3 6JJ UK; 11 Division of Population Health, Health Services Research, and Primary Care, University of Manchester, Oxford Road, Manchester, M139PT UK; 12 Warwick Medical School, University of Warwick, Coventry, UK; 13 Institute of Biomedicine, University of Turku, Kiinamyllynkatu 10, 20014 Turku, Finland; 14 Department of Medical Genetics, Genomics, Laboratory Division, Turku University Hospital, PO Box 52, 20521 Turku, Finland; 15 Department of Applied Health Research, University College London, London, UK; 16 Centre for Cancer Genetic Epidemiology, Department of Oncology, University of Cambridge, Strangeways Laboratory, Worts Causeway, Cambridge, CB1 8RN UK; 17 Department of Applied Health Research, University College London, London, WC1E 7HB UK; 18 Nuffield Department of Surgical Sciences, University of Oxford, Room 6603, Level 6, John Radcliffe Hospital, Headley Way, Headington, Oxford, OX3 9DU UK; 19 Department of Oncology, University of Cambridge, Addenbrooke’s Hospital, Hills Road, Box 279, Cambridge, CB2 0QQ UK; 20 Health and Medical Sciences, University of Copenhagen, 2200 Copenhagen, Denmark; 21 Department of Clinical Biochemistry, Herlev and Gentofte Hospital, Copenhagen University Hospital, Herlev, 2200 Copenhagen, Denmark; 22 Department of Medical Epidemiology and Biostatistics, Karolinska Institute, Stockholm, Sweden; 23 Cancer Epidemiology Division, Cancer Council Victoria, 615 St Kilda Road, Melbourne, VIC 3004 Australia; 24 Centre for Epidemiology and Biostatistics, Melbourne School of Population and Global Health, The University of Melbourne, Grattan Street, Parkville, VIC 3010 Australia; 25 Precision Medicine, School of Clinical Sciences at Monash Health, Monash University, Clayton, VIC 3168 Australia; 26 Center for Genetic Epidemiology, Department of Preventive Medicine, Keck School of Medicine, University of Southern California/Norris Comprehensive Cancer Center, Los Angeles, CA 90015 USA; 27 Cancer Epidemiology Unit, Nuffield Department of Population Health, University of Oxford, Oxford, OX3 7LF UK; 28 Division of Public Health Sciences, Fred Hutchinson Cancer Research Center, Seattle, WA 98109-1024 USA; 29 Department of Epidemiology, School of Public Health, University of Washington, Seattle, WA 98195 USA; 30 Division of Urologic Surgery, Brigham and Womens Hospital, 75 Francis Street, Boston, MA 02115 USA; 31 Department of Genetics and Pathology, International Hereditary Cancer Center, Pomeranian Medical University, Szczecin, Poland; 32 Clinical Gerontology Unit, University of Cambridge, Cambridge, CB2 2QQ UK; 33 Humangenetik Tuebingen, Paul-Ehrlich-Str 23, 72076 Tuebingen, Germany; 34 Department of Laboratory Medicine and Pathology, Mayo Clinic, Rochester, MN 55905 USA; 35 Department of Genetics, Portuguese Oncology Institute of Porto (IPO-Porto), Porto, Portugal; 36 Biomedical Sciences Institute (ICBAS), University of Porto, Porto, Portugal; 37 Division of Epidemiology, Department of Internal Medicine, University of Utah School of Medicine, Salt Lake City, UT USA; 38 George E. Wahlen Department of Veterans Affairs Medical Center, Salt Lake City, UT 84148 USA; 39 Division of Clinical Epidemiology and Aging Research, German Cancer Research Center (DKFZ), 69120 Heidelberg, Germany; 40 German Cancer Consortium (DKTK), German Cancer Research Center (DKFZ), 69120 Heidelberg, Germany; 41 Division of Preventive Oncology, German Cancer Research Center (DKFZ) and National Center for Tumor Diseases (NCT), Im Neuenheimer Feld 460, 69120 Heidelberg, Germany; 42 Department of Medical Chemistry and Biochemistry, Molecular Medicine Center, Medical University of Sofia, Sofia, 2 Zdrave Str., 1431 Sofia, Bulgaria; 43 University of Surrey, Guildford, GU2 7XH Surrey UK; 44 Translational Research Institute, Brisbane, QLD 4102 Australia; 45 Australian Prostate Cancer Research Centre-Qld, Institute of Health and Biomedical Innovation and School of Biomedical Sciences, Queensland University of Technology, Brisbane, QLD 4059 Australia

**Keywords:** Cancer genetics, Genetic interaction, Genotype

## Abstract

Risk classification for prostate cancer (PCa) aggressiveness and underlying mechanisms remain inadequate. Interactions between single nucleotide polymorphisms (SNPs) may provide a solution to fill these gaps. To identify SNP–SNP interactions in the four pathways (the angiogenesis-, mitochondria-, miRNA-, and androgen metabolism-related pathways) associated with PCa aggressiveness, we tested 8587 SNPs for 20,729 cases from the PCa consortium. We identified 3 *KLK3* SNPs, and 1083 (*P* < 3.5 × 10^–9^) and 3145 (*P* < 1 × 10^–5^) SNP–SNP interaction pairs significantly associated with PCa aggressiveness. These SNP pairs associated with PCa aggressiveness were more significant than each of their constituent SNP individual effects. The majority (98.6%) of the 3145 pairs involved *KLK3*. The 3 most common gene–gene interactions were *KLK3-COL4A1:COL4A2*, *KLK3-CDH13,* and *KLK3-TGFBR3.* Predictions from the SNP interaction-based polygenic risk score based on 24 SNP pairs are promising. The prevalence of PCa aggressiveness was 49.8%, 21.9%, and 7.0% for the PCa cases from our cohort with the top 1%, middle 50%, and bottom 1% risk profiles. Potential biological functions of the identified *KLK3* SNP–SNP interactions were supported by gene expression and protein–protein interaction results. Our findings suggest *KLK3* SNP interactions may play an important role in PCa aggressiveness.

## Introduction

Prostate cancer (PCa) accounts for over 10% of all cancer-related deaths, making it the second leading cause of cancer-related deaths among American men in 2021^1^. Because of the substantial clinical heterogeneity of this disease, physicians often have difficulty distinguishing at the time of diagnosis between patients who will develop indolent tumors and those who will develop aggressive PCa^2^. For PCa patients considered to be at a low risk for aggressive PCa, conservative management and treatment are presently recommended. However, ~ 20% of PCa patients who are classified as a low risk using the known classification features (such as prostate specific antigen [PSA], tumor stage, and Gleason score) still die during conservative treatment^3^. This demonstrates an unmet need to identify better biomarkers for predicting PCa aggressiveness.

Genetic association studies have primarily focused on the effects of individual single-nucleotide polymorphisms (SNPs), which are insufficient to explain the complexity of disease susceptibility. However, the majority of SNPs identified by genome-wide association studies (GWAS) are for PCa risk, and only a few are for PCa progression. In fact, only 41 SNPs have been suggested to be associated with PCa progression related phenotypes (such as aggressiveness, early-onset, and PCa survival) in GWAS from the GWAS catalog^4^. These 41 SNPs are located across 53 genes (some are intergenic SNPs), including *KLK3*, *ADGRG1*, *ARHGAP6*, *CASC8,* and *TCF4*^4^. We and others reported several polygenic risk scores (PRS) for PCa risk based on multiple individual SNP effects^5–7^, but the prediction model of PCa aggressiveness remains underdeveloped.

It has been established that gene–gene/SNP–SNP interactions may play a larger role in the causality of complex diseases^8^. Although SNP–SNP interactions have received more attention in the past decade, few have been validated, and most are without known biological functions. The limited SNP–SNP interaction findings may be due to insufficient statistical methods. The conventional approach for testing 2-way SNP–SNP interactions is the Additive–Additive full interaction (AA_Full) approach, the full or hierarchical interaction model (2 main effects + interaction) with the additive SNP inherited mode. AA_Full is the most complicated interaction pattern with the 9 distinct risk-groups, so a large sample size will be needed for detecting this complicated pattern. Using AA_Full to detect SNP–SNP interactions tends to lead to false-negative findings because it only tested one complicated interaction pattern. In order to overcome this issue, we developed two statistical methods: SNP Interaction Pattern Identifier (SIPI) and Additive-Additive 9 Interaction-Model approach (AA9int)^9,10^. The AA9int approach, which treats all SNPs in an additive inheritance mode, tests nine interaction patterns of pairwise SNP–SNP interactions associated with an outcome^10^. The SIPI approach is an extended version of AA9int and tests 45 biologically meaningful SNP–SNP interaction patterns by considering three SNP inheritance modes (additive, dominant and recessive)^9^.

Accumulating evidence suggests that interplay among angiogenesis-, mitochondria-, miRNA-, and androgen metabolism-related pathways may play a critical role in PCa^11–14^. Androgen expression, epigenetic factors, and oxygen levels in the tumor microenvironment regulate angiogenesis, which leads to metastatic PCa^15,16^. Therapies targeting both angiogenesis and androgen for recurrent PCa patients could increase survival rates^17^. These findings suggest that the relationship between androgen and angiogenesis and how their interaction can impact on PCa aggressiveness. Genes involved in androgen metabolism pathways also lead to oncogenic metabolic phenotypes, such as mitochondrial respiration and cell proliferation, in PCa cells. In addition, androgen repression in PCa cells decreases mitochondrial activity^18^. We and others have reported that miRNAs (such as miR-221, miR-222, and miR-155) are involved in regulating various aspects of angiogenesis and PCa progression^12,19^. This evidence suggests that the interplay among genes in these 4 pathways may impact PCa aggressiveness.

This study was inspired by the shortage of SNP findings associated with PCa aggressiveness. Only a small number of SNPs were identified to be associated with PCa aggressiveness, and there is no PRS for PCa aggressiveness. In addition, most of the PRS is based on the sum of several individual SNP effects without considering SNP–SNP interactions. Because of the inspiration, this study's objective is to identify SNP–SNP/gene–gene interactions and to build a SNP-interaction based PRS (SNP_int_-PRS) associated with PCa aggressiveness. These SNPs were selected based on genes in the four PCa biological related pathways (angiogenesis, mitochondria, miRNA, and androgen metabolism).

## Results

### Individual SNP effects

**Figure 1:**
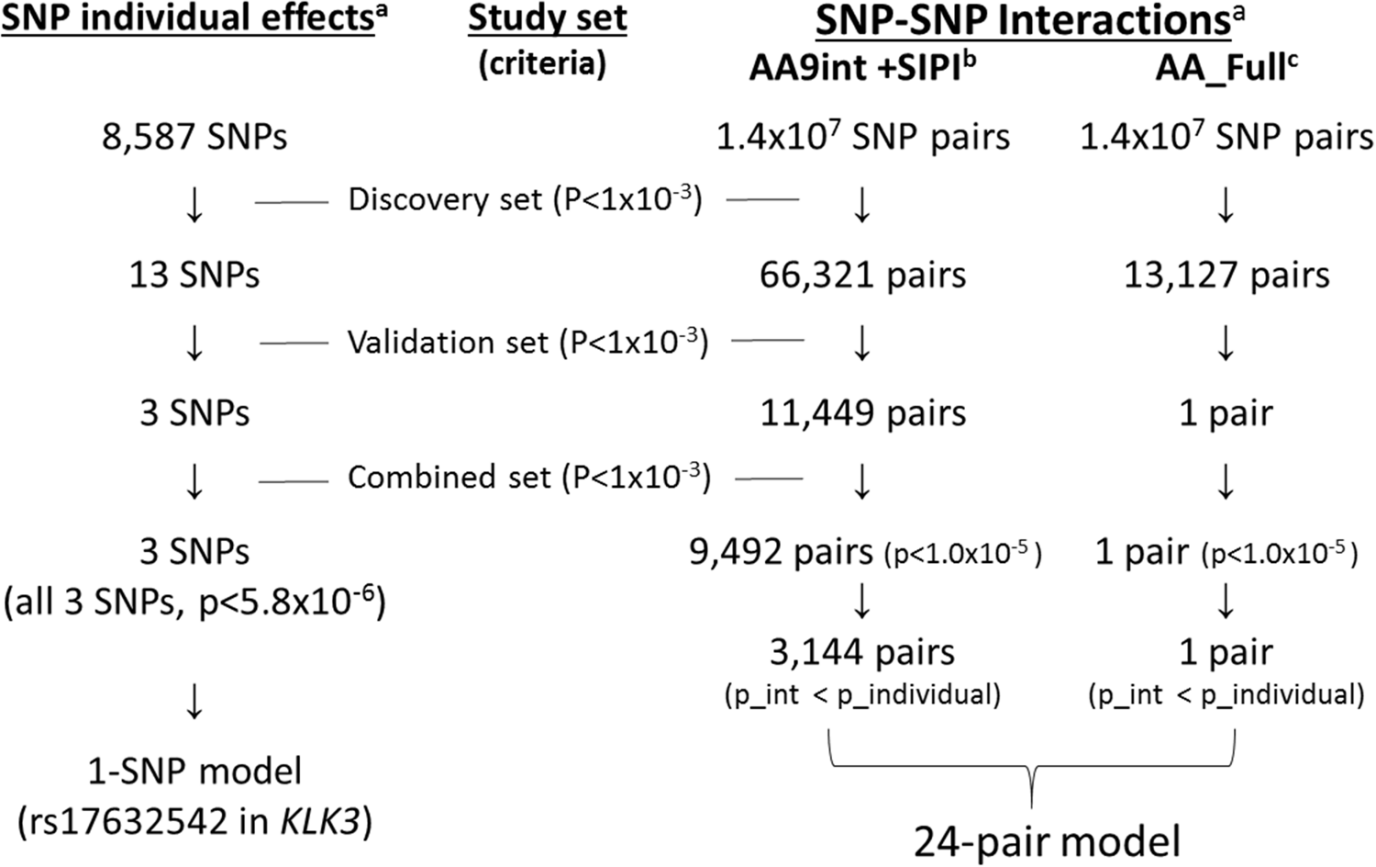
Selection procedure of SNPs and SNP–SNP interaction pairs associated with prostate cancer aggressiveness. ^a^*P* < 5.8 × 10^–6^ is based on Bonferroni correction; candidate SNPs for interaction analyses: 5345 SNPs; 3 SNPs are rs17632542, rs62113212, and rs2569735 in *KLK3*. ^b^For SNP interactions, the 2-stage AA9int + SIPI was applied. Using AA9int, 66,619 pairs were selected with a *P* < 10^–3^ in the discovery set, then 66,321 pairs were selected in the second stage using the SIPI approach. SIPI was applied for the validation and combined set analyses. *AA9int* Additive–Additive 9 interaction-model approach, *p_individual* p-value of individual SNP effect, *p_int* p-value of interaction, *SIPI* SNP interaction pattern identifier, *SNP* single nucleotide polymorphism. ^*c*^*AA_Full* Additive–additive full interaction model.

The process of identifying the significant effects of individual SNPs and SNP–SNP interactions associated with PCa aggressiveness is described in Fig. 1. The individual SNP effect analyses were performed for both the discovery and validation sets. The criterion of a *P* < 0.001 was applied in both the discovery and validation sets to declare validated results. As shown in Table 1, there were 3 *KLK3* SNPs (rs17632542, rs62113212, and rs2569735) with a *P* < 0.001 in both study sets, and all of these three SNPs reached the Bonferroni significance level (*P* < 1.9 × 10^–6^) in the combined set. The best inheritance mode for these 3 SNPs was an additive mode. PCa cases with the minor G allele in rs17632542 tended to have a higher risk of PCa aggressiveness (odds ratio [OR]= 1.45; *P* = 1.7 × 10^–13^). Cases with the minor A allele in rs62113212 (OR per A allele 1.45; *P* = 2.1 × 10^–13^) and rs2569735 (OR= 1.23; *P* = 1.4 × 10^–8^) also had a higher risk of PCa aggressiveness. The relationships for these *KLK3* SNPs are shown in Supplementary Fig. S1. rs17632542 and rs62113212 were highly correlated (linkage disequilibrium [LD] r^2^ = 0.98), and the association between rs17632542 and rs2569735 was moderate (LD r^2^ = 0.42).

**Table 1:** Individual SNP effects associated with prostate cancer aggressiveness.

SNP^a^	Min < Maj (MAF)	Combined set			Discovery set	Validation set	Genes
		*P* value^b^	OR (95% CI)^b^	Mode	*P* value^b^	*P* value^b^	
rs17632542	G < A (0.06)	1.7 × 10^–13^	1.45 (1.31, 1.60)	Add	5.8 × 10^–8^	1.3 × 10^–7^	*KLK3*
rs62113212	A < G (0.06)	2.1 × 10^–13^	1.45 (1.31, 1.59)	Add	1.4 × 10^–7^	7.3 × 10^–8^	*KLK3*
rs2569735	A < G (0.12)	1.4 × 10^–8^	1.23 (1.14, 1.32)	Add	9.3 × 10^–5^	2.7 × 10^–5^	*KLK3*
rs1058205	G < A (0.15)	5.0 × 10^–7^	1.18 (1.11, 1.26)	Add	1.6 × 10^–3^	5.3 × 10^–5^	*KLK3*
rs174776	A < G (0.11)	4.9 × 10^–6^	1.19 (1.10, 1.28)	Add	2.3 × 10^–3^	4.7 × 10^–4^	*KLK3*
rs266876	G < A (0.24)	1.2 × 10^–5^	1.13 (1.07, 1.20)	Add	2.0 × 10^–2^	8.1 × 10^–5^	*KLK3*
rs2271095	G < A (0.35)	1.7 × 10^–5^	0.90 (0.85, 0.94)	Add	3.1 × 10^–3^	2.0 × 10^–3^	*KLK3*
rs4802755	A < G (0.46)	2.0 × 10^–5^	1.11 (1.06, 1.17)	Add	2.0 × 10^–2^	8.0 × 10^–5^	*KLK3*
rs7446	A < G (0.31)	2.4 × 10^–5^	1.16 (1.08, 1.24)	Dom	3.9 × 10^–2^	9.1 × 10^–5^	*KPNA3*
rs6998	A < G (0.37)	6.3 × 10^–5^	0.90 (0.86, 0.95)	Add	9.2 × 10^–3^	2.3 × 10^–3^	*KLK3*
rs4802754	A < G (0.29)	1.8 × 10^–4^	0.90 (0.86, 0.95)	Add	1.9 × 10^–3^	3.0 × 10^–2^	*KLK3*
rs2361634	G < A (0.07)	3.5 × 10^–4^	1.12 (1.05, 1.20)	Add	2.9 × 10^–2^	5.0 × 10^–3^	*AR*

*add* additive, *dom* dominant, *MAF* minor allele frequency, *maj* major allele, *min* minor allele, *rec* recessive, *SNP* single nucleotide polymorphism.

^a^First 3 SNPs are validated SNPs (discovery p < 1 × 10^–3^ and validation p < 1 × 10^–3^). Except rs62113212, other SNPs are the hub SNPs in the 11 clusters. Linkage disequilibrium: r^2^(rs17632542, rs62113212) = 0.98.

^b^All models adjusted for study sites and six principal components for population stratification; *OR* odds ratio, *CI* confidence interval.

### SNP–SNP interactions

By applying the AA_Full approach for 1.4 × 10^7^ SNP pairs associated with PCa aggressiveness, only one pair (rs390993 + rs473640 in *RIPK2* and *NOS1*) qualified the selection criteria: P < 0.001 in the discovery, validation, and combined sets (Fig. 1). Using the AA_Full approach, 13,127 SNP pairs had P < 0.001 in the discovery set. Among them, only 1 pair (rs390993 + rs473640 in *RIPK2* and *NOS1*) satisfied the validation criteria. The interaction p-values of rs390993 + rs473640 were 4.5 × 10^–5^, 9.2 × 10^–4^, and 2.8 × 10^–7^ for the discovery, validation, and combined sets. The interaction patterns for this SNP pair were shown in Supplementary Fig. S2.

We applied the 2-stage Additive–Additive 9 interaction model approach and SNP Interaction Pattern Identifier (AA9int + SIPI) approach to search for SNP–SNP interactions associated with PCa aggressiveness in the discovery set to reduce computation burden and maintain prediction power (Fig. 1). When testing 1.4 × 10^7^ SNP pairs using the AA9int approach, 66,619 pairs had a *P* < 0.001. Then, we applied SIPI for further interaction analyses and found 66,321 pairs with a *P* < 0.001 in the discovery set, of which 11,449 pairs were validated. In the combined set, 9492 pairs were promising because they had a P < 1 × 10^–5^, which was the criterion we selected based on the plot of the − log10 P values in Supplementary Fig. S3. Further, 3795 of these SNP pairs also met the stringent Bonferroni criterion (*P* < 3.5 × 10^–9^). Among 3795 SNP pairs, 1083 pairs’ interaction had a lower p-value than their 2 constituent SNP effects. Of the 9492 promising pairs, 3144 SNP interaction pairs were more significant than their constituent SNPs. All 1083 SNP pairs and 98.6% of the top 3144 promising pairs associated with PCa aggressiveness were involved with *KLK3* SNPs. Among the top 3144 SNP pairs associated with PCa aggressiveness, 4 *KLK3* clusters contributed 91% of these pairs (n = 2856/3144). These 4 *KLK3* clusters were rs17632542 (769 pairs), rs2569735 (1132 pairs), rs1058205 (601 pairs), and rs174776 (350 pairs). The most significant 5 SNP pairs within these 4 *KLK3* clusters are listed in Table 2, and the top non-*KLK3* pairs with *P* < 1 × 10^–6^ are listed in Supplementary Table S1.

**Table 2:** Top 5 pairs for the four KLK3 clusters of rs2569735, rs17632542, rs1058205, and rs174776 associated with prostate cancer aggressiveness.

SNP1	SNP2	Interaction pattern label^a^	Pattern details	SNP1 Min < Maj (MAF)	SNP2 Min < Maj (MAF)	Combined *P* value^b^	OR (95% CI)^b^	Dis. *P* value^b^	Val. *P* value^b^	Gene1	Gene2
rs4783709	rs17632542	AA_int_ro	SNP1xSNP2 (G,G)	A < G (0.31)	G < A (0.06)	4.6 × 10^–16^	1.29 (1.21, 1.37)	2.2 × 10^–8^	3.1 × 10^–9^	*CYB5B: LOC105371325*	*KLK3*
rs2050143	rs17632542	AA_int_ro	SNP1xSNP2 (A,G)	G < A (0.28)	G < A (0.06)	6.7 × 10^–16^	1.29 (1.21, 1.37)	3.9 × 10^–10^	1.6 × 10^–8^	*PDGFB*	*KLK3*
rs16837637	rs17632542	RD_int_ro	(GG/GA + AG/GG) vs. others	A < G (0.39)	G < A (0.06)	6.8 × 10^–16^	1.57 (1.41, 1.75)	1.1 × 10^–7^	5.5 × 10^–10^	*NRP2*	*KLK3*
rs9301460	rs17632542	AA_int_ro	SNP1xSNP2 (G,G)	A < G (0.38)	G < A (0.06)	1.2 × 10^–15^	1.31 (1.23, 1.40)	9.0 × 10^–10^	1.6 × 10^–8^	*COL4A2:COL4A2-AS1*	*KLK3*
rs7196117	rs17632542	RD_int_rr	(AA/AG + AA) vs. others	G < A (0.19)	G < A (0.06)	1.9 × 10^–15^	0.69 (0.63, 0.76)	1.0 × 10^–8^	2.3 × 10^–8^	*LOC105371286: LOC105371287*	*KLK3*
rs17632542	rs2569735	DD_int_oo	(AG/GG + GA/AA) vs. others	G < A (0.06)	A < G (0.12)	5.2 × 10^–13^	1.46 (1.32, 1.62)	5.3 × 10^–8^	7.2 × 10^–8^	*KLK3*	*KLK3*
rs7613553	rs2569735	AA_int_ro	SNP1xSNP2 (C,A)	A < C (0.44)	A < G (0.121)	7.2 × 10^–12^	1.20 (1.14, 1.26)	3.0 × 10^–7^	3.2 × 10^–6^	*RARB*	*KLK3*
rs2292185	rs2569735	AA_int_ro	SNP1xSNP2 (G,A)	A < G (0.39)	A < G (0.12)	3.6 × 10^–11^	1.15 (1.10, 1.20)	5.5 × 10^–6^	9.3 × 10^–7^	*KLK3*	*KLK3*
rs2569735	rs4802754	DD_int_or	(GA + AA/GG) vs. others	A < G (0.12)	A < G (0.29)	4.2 × 10^–11^	1.36 (1.24, 1.49)	2.6 × 10^–6^	1.5 × 10^–6^	*KLK3*	*KLK3*
rs2569735	rs2766535	AA_M2_int_r2	SNP2 (G) SNP1xSNP2 (A,G)	A < G (0.12)	A < G (0.45)	0.001 5.3 × 10^–11^	0.92 (0.87, 0.97) 1.20 (1.13, 1.26)	3.4 × 10^–7^	5.2 × 10^–6^	*KLK3*	*FKBP5*
rs1058205	rs17632542	DD_int_oo	(AG/GG + AG/GG) vs. others	G < A (0.15)	G < A (0.06)	5.9 × 10^–13^	1.46 (1.32, 1.61)	1.3 × 10^–7^	1.7 × 10^–7^	*KLK3*	*KLK3*
rs1058205	rs2361634	AA_int_rr	SNP1xSNP2 (A,A)	G < A (0.15)	G < A (0.07)	5.2 × 10^–10^	0.92 (0.90, 0.95)	1.2 × 10^–4^	7.1 × 10^–7^	*KLK3*	*AR*
rs385037	rs1058205	AA_int_ro	SNP1xSNP2 (A,G)	G < A (0.41)	G < A (0.15)	1.5 × 10^–9^	1.15 (1.10, 1.20)	3.8 × 10^–5^	7.0 × 10^–6^	*RAB20*	*KLK3*
rs7613553	rs1058205	AA_int_ro	SNP1xSNP2 (C,G)	A < C (0.44)	G < A (0.15)	1.5 × 10^–9^	1.15 (1.10, 1.21)	1.6 × 10^–5^	1.6 × 10^–5^	*RARB*	*KLK3*
rs2274545	rs1058205	AA_int_ro	SNP1xSNP2 (A,G)	C < A (0.28)	G < A (0.15)	2.0 × 10^–9^	1.13 (1.09, 1.18)	7.4 × 10^–5^	4.7 × 10^–6^	*COL4A2*	*KLK3*
rs174776	rs17632542	RD_int_rr	(GG/GA + AA) vs. others	A < G (0.11)	G < A (0.06)	1.6 × 10^–14^	0.68 (0.61, 0.75)	3.0 × 10^–8^	6.3 × 10^–8^	*KLK3*	*KLK3*
rs10520259	rs174776	AA_M1_int_o1	SNP1(A) SNP1xSNP2 (A,A)	A < G (0.28)	A < G (0.11)	0.001 1.2 × 10^–9^	0.91 (0.86, 0.96) 1.31 (1.20, 1.43)	5.7 × 10^–5^	6.4 × 10^–4^	*HAND2*	*KLK3*
rs1250240	rs174776	DR_int_ro	(GG + AA) vs. others	A < G (0.26)	A < G (0.11)	2.4 × 10^–9^	2.96 (2.07, 4.24)	5.9 × 10^–5^	1.0 × 10^–5^	*FN1*	*KLK3*
rs174776	rs2361634	AA_int_rr	SNP1xSNP2 (G,A)	A < G (0.11)	G < A (0.07)	4.3 × 10^–9^	0.92 (0.90, 0.95)	1.1 × 10^–4^	8.9 × 10^–6^	*KLK3*	*AR*
rs174776	rs2569735	AA_int_oo	SNP1xSNP2 (A,A)	A < G (0.11)	A < G (0.12)	7.9 × 10^–9^	1.21 (1.14, 1.29)	3.1 × 10^–5^	4.0 × 10^–5^	*KLK3*	*KLK3*

*CI* confidence interval, *Dis* discovery set, *MAF* minor allele frequency, *Maj* major allele, *Min* minor allele, *OR* odds ratio, *Val* validation set.

^a^SIPI pattern interpretation: The first 2 letters represent inheritance modes of the first SNP (or SNP1) and the 2nd SNPs (or SNP2); mode (A, additive mode; D, dominant mode; R, recessive mode; “(SNP1 mode)(SNP2 mode)_int_(SNP1 code)(SNP2 code)” is for interaction-only pattern; “Mϕ_int_”, interaction plus one main effect of SNPϕ; SNP code (o, original coding based on the minor allele; r, reverse coding; o1: original coding for SNP1 (SNP2 with original coding); r2: reverse coding of SNP2 (SNP1 with original coding).

^b^For verified SNP pairs with P < 1 × 10^–5^ in the combined set and p_interaction < p_individual; all models adjusted for study site and first six principal components.

**Figure 2:**
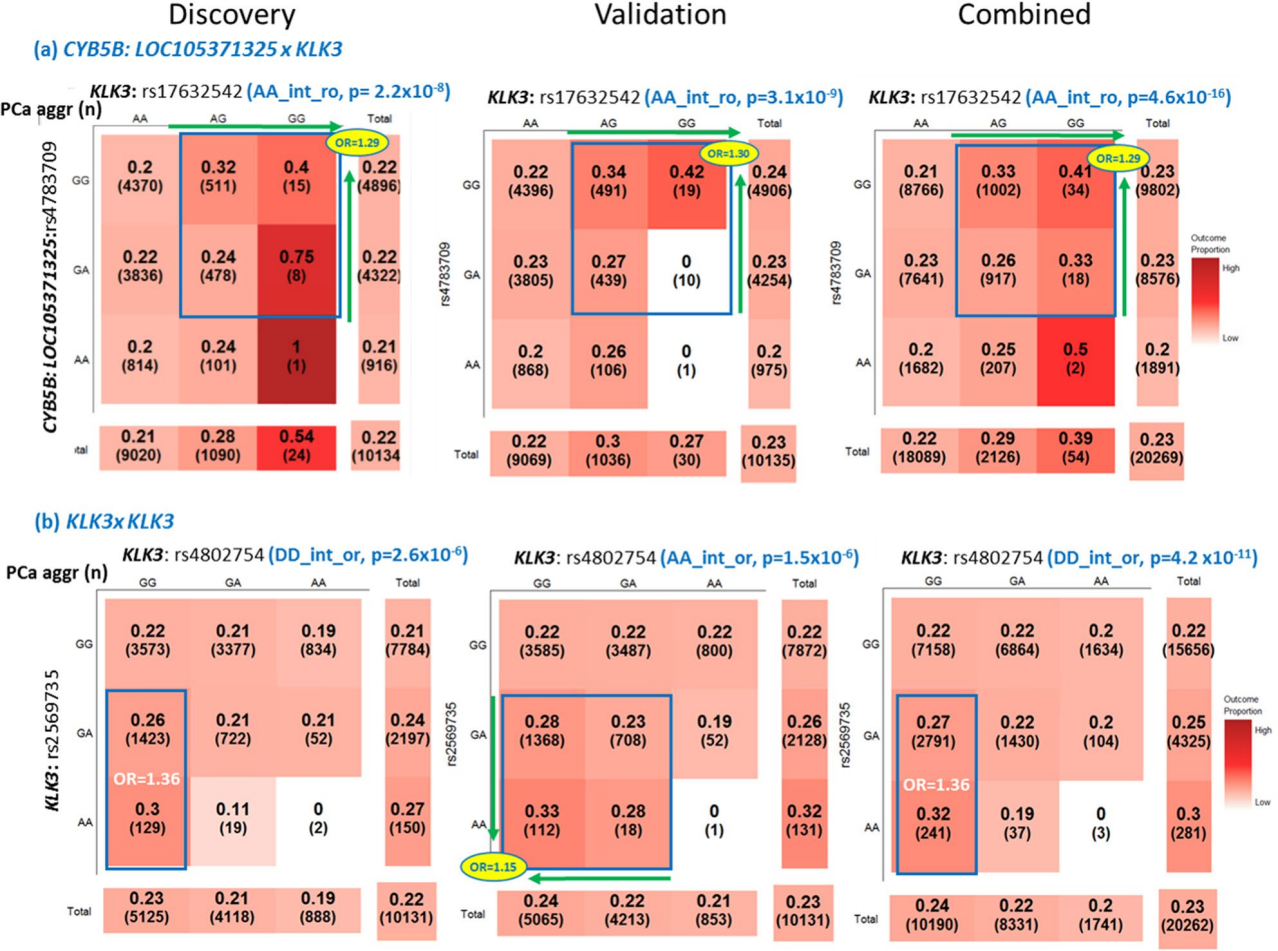
Selected SNP–SNP interactions associated with prostate cancer aggressiveness. PCa aggr (n): prevalence of prostate cancer aggressiveness (sample size in the genotype combination); SNP–SNP Interaction Pattern: “(SNP1 mode)(SNP2 mode)**_int_**(SNP1 code)(SNP2 code)”; OR of PCa aggressiveness adjusted for study sites and six principal components for population stratification. The darker color indicates a higher chance of prostate cancer aggressiveness. *A* additive inheritance modes, *aggr* aggressiveness, *D* dominant inheritance modes, *o* original coding based on the minor allele, *OR* odds ratio, *PCa* prostate cancer, *R* recessive inheritance modes, *r* reverse code direction, *SNP* single-nucleotide polymorphism. These heat tables were generated using the plot3by3 function in the SIPI R package.

The most significant SNP pair was rs4783709 + rs17632542 in *CYB5B: LOC105371325* and *KLK3,* with a *P* value of 4.6 × 10^–16^ in the combined set, 2.2 × 10^–8^ in the discovery set, and 3.1 × 10^–9^ in the validation set. As shown in Table 2 and Fig. 2A, the identified interaction pattern for the rs4783709 + rs17632542 SNP pair in the discovery, validation, and combined sets was *AA_int_ro*, which indicates the additive–additive interaction-only pattern with the reverse and original mode for the first and second SNPs, respectively. PCa cases with the major allele G in rs4783709 and the minor allele G in rs17632542 in *KLK3* had a higher risk of PCa aggressiveness (*P* = 4.6 × 10^–16^; OR= 1.29 per one unit of the G × G allele with a coding of 0, 1, 2 for each allele) in the combined set. In the SNP pair of rs4783709 + rs17632542, the prevalence of PCa aggressiveness was 20%, 26%, 33%, and 41% for PCa cases with the genotype combinations of AA + AA, GA + AG, GG/AG, and GG + GG, respectively, with a respective coding of 0, 1, 2, and 4. The rs2569735 + rs4802754 (2 *KLK3* SNP interactions) pair in the combined set (Fig. 2B) had a *DD_int_or* pattern, which indicates a dominant–dominant interaction-only pattern with the original mode for SNP1 and reverse mode for SNP2. The PCa cases with the *GA/AA* + *GG* genotype in rs2569735 + rs4802754, respectively, had a higher risk of PCa aggressiveness (OR= 1.36; *P* = 4.2 × 10^–11^) compared with the group with other genotypes for this SNP pair. As shown in Fig. 2B, the prevalence of PCa aggressiveness was 23% for the entire cohort and 27–32% for the *GA/AA* + *GG* group in the SNP pair of rs2569735 + rs4802754. Although the validation set had a different interaction pattern of *AA_int_or* compared with the discovery and combined sets, their trends were similar.

The majority of the identified SNP interactions are interaction-only models with only 1 interaction term in each model; only a few SNP interactions had a complicated model with > 1 terms (such as *M1_int* or *M2_int*). Some examples of these complicated interaction patterns are listed in Table 2 and Supplementary Fig. S4. The interaction pattern for the SNP pair of rs10520259 in *HAND2* + rs174776 in *KLK3,* which was associated with PCa aggressiveness, was *AA_M1_int_o1*. This is an additive–additive model plus the main effect of rs10520259 (rs10520259 + rs10520259 × rs174776) with an original mode for both SNPs. Among PCa cases with the rs174776 *GG* genotype, an A allele of rs10520259 was associated with a lower risk of PCa aggressiveness (OR= 0.91; *P* = 0.001). For PCa cases with the rs174776 *GA/AA* genotypes, the rs10520259 A allele had an opposite effect (OR= 1.31; *P* = 1.2 × 10^–9^).

### Examples of SNP–SNP interaction pairs with a large effect size

There were 8 significant SNP pairs with a large effect size (OR ≥ 2.00) associated with PCa aggressiveness for the binary-mode interaction models, including the *DD_*, *DR_*, *RD_*, and *RR_* models. To identify a large effect size, different OR criteria should be applied for the binary-mode interaction models and AA models because they have different coding scales: (0, 1) for the binary-mode interaction models and (0, 1, 2, and 4) for the AA models. For AA models, there were 20 pairs with OR ≥ 1.3, and the top 5 pairs with OR ≥ 1.35 are shown in Table 3. All 5 of these pairs are involved with *KLK3* rs17632542.

**Table 3:** SNP interaction pairs with a large effect size associated with prostate cancer aggressiveness.

SNP1	SNP2	Interaction pattern label^a^	Pattern details	SNP1 Min < Maj (MAF)	SNP2 Min < Maj (MAF)	Combined *P* value^b^	OR (95% CI)^b^	Dis. *P* value^b^	Val. *P* value^b^	Gene1	Gene2
rs3775202	rs174776	RR_int_oo	(AA + AA) vs. others	A < G (0.49)	A < G (0.11)	5.9 × 10^–7^	4.12 (2.36, 7.19)	1.2 × 10^–4^	2.3 × 10^–4^	*VEGFC*	*KLK3*
rs2317676	rs7802277	DR_M2_int_o2	(AA + AA) (AG/GG + AA) vs. others	G < A (0.07)	A < G (0.14)	8.3 × 10^–4^ 6.5 × 10^–5^	0.62 (0.47, 0.82) 3.13 (1.79, 5.47)	9.2 × 10^–4^	9.2 × 10^–4^	*ITGB3*	*MTCYBP42*
rs1250240	rs174776	DR_int_ro	(GG + AA) vs. others	A < G (0.26)	A < G (0.11)	2.4 × 10^–9^	2.96 (2.07, 4.24)	5.8 × 10^–5^	1.0 × 10^–5^	*FN1*	*KLK3*
rs7224135	rs174776	DR_int_ro	(GG + AA) vs. others	A < G (0.42)	A < G (0.11)	9.5 × 10^–7^	2.88 (1.89, 4.40)	7.0 × 10^–4^	3.8 × 10^–4^	*CAVIN1*	*KLK3*
rs2075756	rs174776	DR_int_ro	(GG + AA) vs. others	A < G (0.28)	A < G (0.11)	4.1 × 10^–8^	2.74 (1.91, 3.92)	1.8 × 10^–4^	4.8 × 10^–5^	*TRIP6*	*KLK3*
rs10467147	rs174776	DR_int_ro	(GG + AA) vs. others	A < G (0.33)	A < G (0.11)	4.0 × 10^–6^	2.50 (1.69, 3.69)	1.6 × 10^–4^	4.4 × 10^–4^	*LRRK2*	*KLK3*
rs1980499	rs174776	RR_int_ro	(AA/AG + AA) vs. others	G < A (0.49)	A < G (0.11)	1.1 × 10^–6^	2.19 (1.60, 3.00)	1.3 × 10^–4^	4.5 × 10^–5^	*BMP2*	*KLK3*
rs2224524	rs174776	RR_int_ro	(GG/GA + AA) vs. others	A < G (0.43)	A < G (0.11)	3.3 × 10^–6^	2.01 (1.50, 2.69)	6.5 × 10^–4^	6.0 × 10^–4^	*LOC107987087: RASEF*	*KLK3*
rs27650	rs17632542	AA_int_oo	SNP1xSNP2 (A,G)	A < G (0.45)	G < A (0.06)	7.1 × 10^–15^	1.39 (1.28, 1.51)	3.0 × 10^–9^	1.9 × 10^–8^	*RASGRF2*	*KLK3*
rs414881	rs17632542	AA_int_oo	SNP1xSNP2 (A,G)	A < G (0.48)	G < A (0.06)	6.5 × 10^–15^	1.36 (1.26, 1.46)	4.1 × 10^–10^	2.3 × 10^–7^	*COL4A2:COL4A2-AS1*	*KLK3*
rs45631565	rs17632542	AA_int_oo	SNP1xSNP2 (A,G)	A < C (0.43)	G < A (0.06)	1.0 × 10^–13^	1.37 (1.26, 1.48)	7.7 × 10^–9^	1.3 × 10^–6^	*FGFR2*	*KLK3*
rs587409	rs17632542	AA_int_oo	SNP1xSNP2 (A,G)	A < G (0.47)	G < A (0.06)	2.3 × 10^–15^	1.38 (1.27, 1.49)	3.0 × 10^–9^	1.6 × 10^–8^	*COL4A1*	*KLK3*
rs9521801	rs17632542	AA_int_ro	SNP1xSNP2 (G,G)	A < G (0.5)	G < A (0.06)	4.5 × 10^–15^	1.36 (1.26, 1.46)	4.8 × 10^–7^	6.2 × 10^–10^	*COL4A2*	*KLK3*

*CI* confidence interval, *Dis* discovery set, *MAF* minor allele frequency, *Maj* major allele, *Min* minor allele, *OR* odds ratio, *Val* validation set.

^a^SIPI pattern interpretation: The first 2 letters represent inheritance modes of the first SNP (or SNP1) and the 2^nd^ SNPs (or SNP2); mode (A, additive mode; D, dominant mode; R, recessive mode; “(SNP1 mode)(SNP2 mode)_int_(SNP1 code)(SNP2 code)” is for interaction-only pattern; “Mϕ_int_”, interaction plus one main effect of SNPϕ;

SNP code (o, original coding based on the minor allele; r, reverse coding; o1: original coding for SNP1 (SNP2 with original coding); r2: reverse coding of SNP2 (SNP1 with original coding).

^b^For verified SNP pairs with P < 1 × 10^–5^ in the combined set and p_interaction < p_individual; all models adjusted for study site and first six principal components.

**Figure 3:**
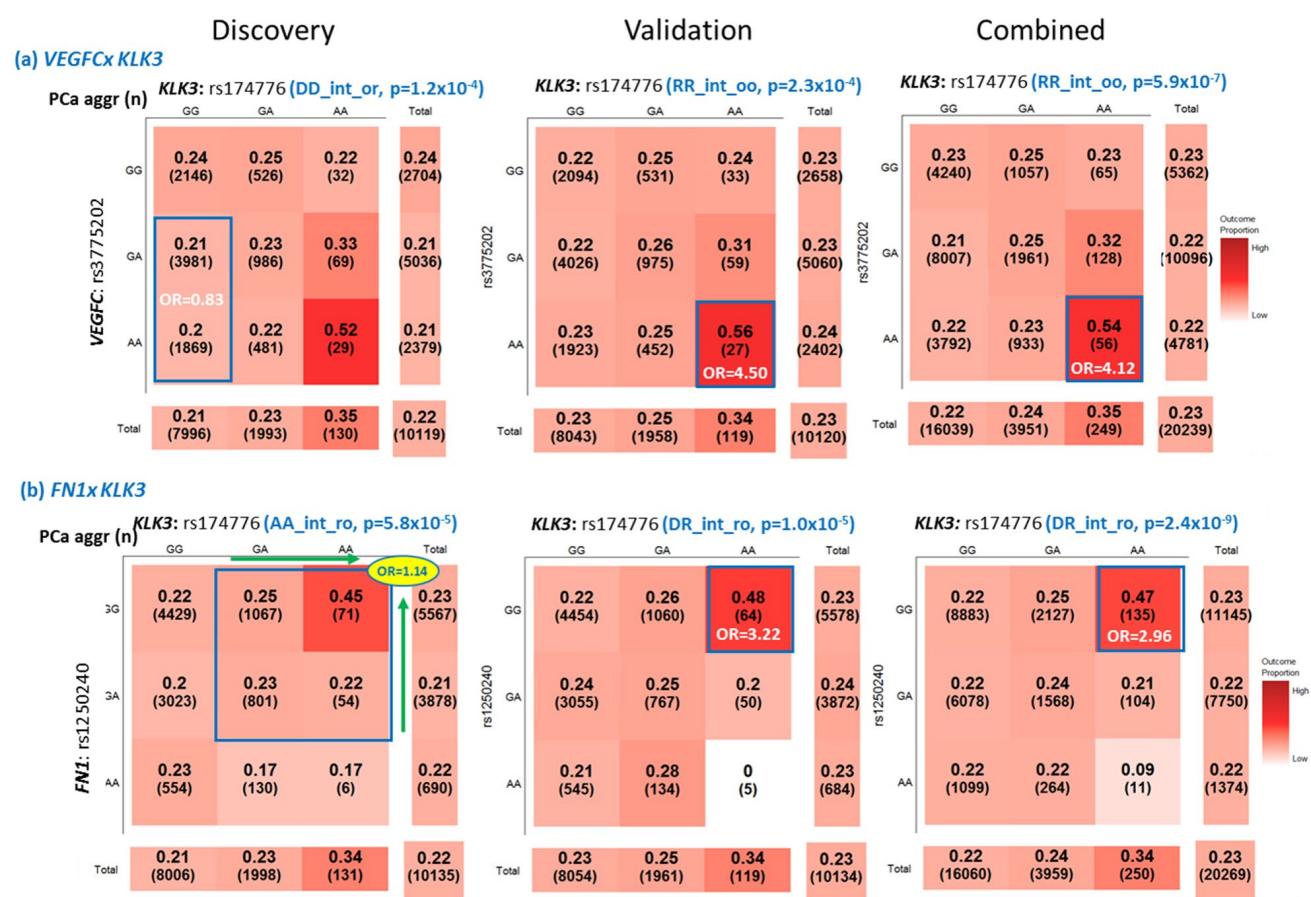
Selected SNP–SNP interactions with a large effect size associated with prostate cancer aggressiveness. Note: PCa aggr (n): prevalence of prostate cancer aggressiveness (sample size in the genotype combination); SNP–SNP Interaction Pattern: “(SNP1 mode)(SNP2 mode)**_int_**(SNP1 code)(SNP2 code)”; OR: odds ratio of PCa aggressiveness adjusted for study sites and 6 principal components for population stratification. The darker color indicates a higher risk of prostate cancer aggressiveness. *A* additive inheritance mode, *aggr* aggressiveness, *D* dominant inheritance mode, *o* original coding based on the minor allele, *OR* odds ratio, *PCa* prostate cancer, *R* recessive inheritance mode, *r* reverse code direction, *SNP* single-nucleotide polymorphism. These heat tables were generated using the plot3by3 function in the SIPI R package.

As shown in Table 3, the pair with the largest effect size was rs3775202 + rs174776 with the *RR_int_oo* pattern, a recessive–recessive interaction-only pattern with an original mode. The interaction pattern in the discovery set was not the same but had a similar trend. Though the prevalence of aggressive PCa for the entire cohort was 23% (Fig. 3A), the prevalence among the cases with the *AA* + *AA* genotype in rs3775202 + rs174776 in *VEGFC* and *KLK3* was 54% (OR= 4.12 for *AA* + *AA* genotype compared with others; *P* = 5.9 × 10^–7^) in the combined set. Another example of an SNP pair with a large effect size was rs1250240 + rs174776 (*FN1* + *KLK3*) with the *DR_int_ro* pattern, a dominant–recessive interaction with a reverse mode in the validation and combined sets (Fig. 3B). PCa cases with the *GG* + *AA* genotype in rs1250240 + rs174776 had a higher risk of being aggressive (OR= 3.22 and 2.96 in the validation and combined sets, respectively). The interaction pattern of this pair was similar in the discovery set. As shown in Fig. 3A,B, PCa prevalence for a specific SNP genotype would be altered when interacting with another SNP. Our results demonstrated that these SNP–SNP interaction patterns could explain the PCa aggressiveness profile better than their constituent SNPs’ individual effects.

The pair with the second largest effect size was rs2317676 + rs7802277 (*ITGB3* + *MTCYBP42*) with the *DR_M2_int_o2* pattern, a dominant–recessive interaction with the main effect of the second SNP (rs7802277) and an original mode for both SNPs. As shown in Table 3 and Supplementary Fig. S4, the prevalence of aggressive PCa was 44% for cases with the *AG/GG* + *AA* genotype (OR= 3.13; *P* = 6.5 × 10^–5^) and only 16% for cases with the *AA* + *AA* genotype (OR= 0.62; *P* = 8.3 × 10^–4^), whereas the overall prevalence of aggressive PCa was 23% in the combined set. In addition, there were 88 SNP pairs associated with PCa aggressiveness, with a medium effect size (0.5 ≤ OR < 0.67 or 1.5 ≤ OR < 2) for SNP pairs with binary modes.

### Non-*KLK3* SNP–SNP interaction pairs

Only 43 SNP pairs among the top 3144 SIPI identified pairs did not involve *KLK3*, and 21 out of these 43 pairs were involved with rs7446 in *KPNA3*. The top 17 SNP pairs with a *P* < 10^–6^ are listed in Supplementary Table S1. For the most significant SNP pair of rs2266967 (*MAPK1*) + rs7446 (*KPNA3*), PCa cases with the *CA/AA* + *AG/AA* genotype had a higher risk of PCa aggressiveness (OR= 1.21; *P* = 3.6 × 10^–8^) compared to cases with other genotypes for this SNP pair. The SNP pair with the largest effect size was rs2317676 (*ITGB3*) + rs7802277 (*MTCYBP42*).

### SNP-interaction PRS

Among the 3144 candidate pairs selected using the AA9int + SIPI approach, the majority were in the 11 clusters identified in Supplementary Table S2; only 23 pairs were not involved in these clusters. Some SNP pairs showed up in 2 clusters (such that SNP_A_–SNP_B_ showed up in both cluster A and cluster B), and so we dropped the duplicated pairs for model building. Nine of these 11 clusters were involved with *KLK3*. By deleting the highly correlated SNP pairs and performing variable selection associated with PCa aggressiveness within each cluster (Supplementary Table S2), the number of candidate pairs for modeling was reduced from 3144 to 96 pairs. The largest cluster was for rs2569735 in *KLK3* with 1132 pairs, of which only 23 were low-correlated pairs (r < 0.7). After applying stepwise selection for pairs associated with PCa aggressiveness with *P* < 0.1 as the selection criteria within a cluster, only 14 SNPs pairs were selected. The number of the selected pairs for other clusters are listed in Supplementary Table S2. The candidate set for the multipair model was composed of 96 pairs, including 76 selected pairs within the 11 clusters and 20 other SNP pairs.

We applied stepwise selection in logistic model for the SIPI selected pairs in the combined set. Using this approach, 23 pairs and 12 pairs were selected based on a criterion of *P* < 0.01 and *P* < 1 × 10^–5^, respectively. We evaluated the 24-pair model by adding 1 SNP pair (rs390993 + rs473640 in *RIPK2* and *NOS1*) selected using the AA_Full approach to the list of 23 SNP pairs. The 24-pair model comprised interactions between 42 SNPs (Supplementary Table S3), and 9 of these pairs were involved with the *KLK3* SNPs. The 12-pair model was made of interactions between 24 total SNPs, and 3 pairs were involved with the *KLK3* SNPs. Based on these 2 models, we built the SNP_int_-PRS, then classified PCa cases into 7 groups based on their risk profiles (see Supplementary Methods).

**Figure 4:**
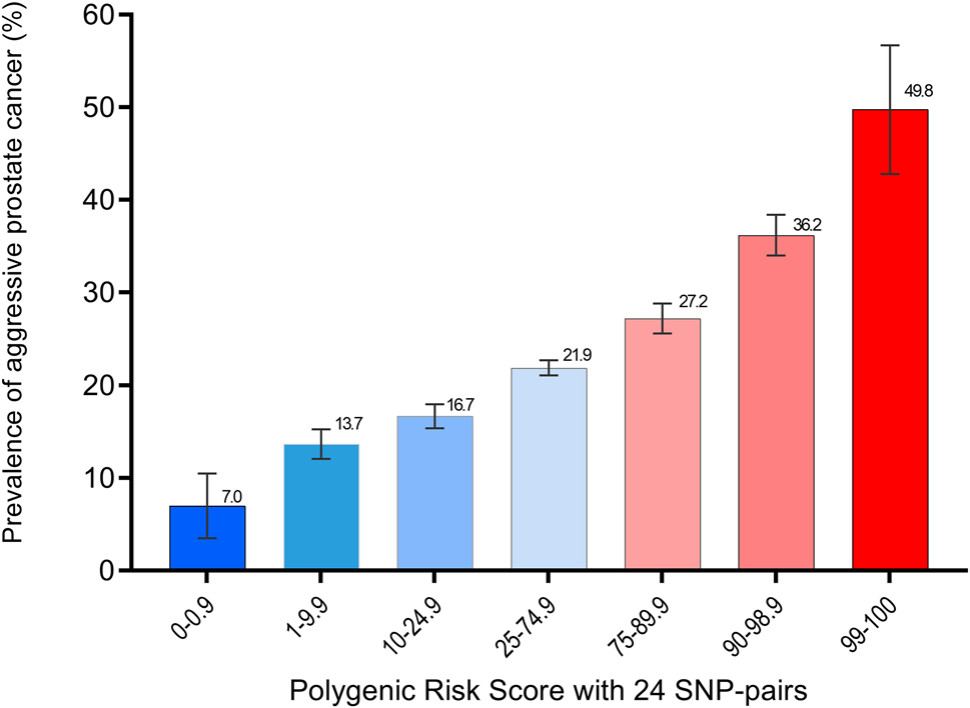
Performance of the polygenic risk score of prostate cancer aggressiveness based on the 24 SNP pairs. *PCa* prostate cancer, *SNP* single-nucleotide polymorphism. Mean and 95% confidence intervals of PCa aggressiveness prevalence were shown.

For the 24-pair model (Supplementary Table S4A), the OR of aggressiveness for PCa cases in the top 1% high-risk group was 3.65 (95% CI= 2.71–4.91), and the OR for cases in the second highest risk group was 1.97 by using the 50% PCa cases with an average risk as to the reference group after adjusting for study site and the 6 principal components of population stratification as suggested by the PRACTICAL study^20^. In addition, the ORs of the cases with the lowest 1% and the 1–10% risk profile were 0.27 and 0.55, respectively. As shown in Fig. 4, the prevalence of PCa aggressiveness for cases with the top 1%, middle 50%, and bottom 1% risk profiles were 49.8%, 21.9%, and 7.0%, respectively. For the 12-pair model (Supplementary Table S4B), the ORs of PCa aggressiveness for the top 1% and bottom 1% were 3.06 and 0.51, respectively.

For the individual-effect model, only rs17632542 was selected based on the stepwise selection with a significance level of 1 × 10^–5^ based on the 3 verified *KLK3* SNPs in Table 1. As shown in Supplementary Table S5, we compared the performance of these 2 SNP_int_-PRS with this individual-effect model using the area under the receiver operator characteristics curve (AUC). The AUC values for the 24-pair model, 12-pair model and individual-effect model were 0.696, 0.687, and 0.668, respectively. The 24-pair model performed significantly better than the individual-effect model with rs17632542 and the 12-pair model (AUC comparison *P* = 6.5 × 10^–27^ and 2.7 × 10^–8^, respectively). We also performed an internal validation using the bootstrap approach. The AUC’s 95% confidence interval for the 24-pair interaction model was 0.686–0.702 based on 1000 bootstrap runs.

### Expression quantitative trait loci analyses

To evaluate the biological functions of the identified SNP pairs, we performed 2-way expression quantitative trait loci (eQTL) tests, which evaluated associations between 1 identified SNP pair (2 SNPs) and 1 gene expression in the 4 target pathways. The linear model-based AA9int approach was applied. As shown in Supplementary Table S5, there were 24 significant eQTL tests with a *P* value < 1.1 × 10^–8^ (Bonferroni criteria = 0.05/4.5 × 10^6^). The distribution of − log10-transformed *P* values for the eQTL tests is shown in Supplementary Fig. S5. Among these 24 significant eQTL tests (Supplementary Table S5), all of them involved 1 of the 4 top *KLK3* SNPs (rs17632542, rs2569735, rs1058205, and rs174776), and 16 tests involved SNP pairs within the top 3144 pairs. One SNP in *FHIT*, rs995633, interacted with 2 *KLK3* SNPs (rs1058205 and rs2569735), which link to *SLC25A21* expression. In addition, the genotype combinations of rs7224135 (*CAVIN1*) + rs174776 (*KLK3*) had a significant effect on *SRD5A2* expression (Supplementary Fig. S6, *P* = 2.5 × 10^–9^). These promising eQTL results support that the *KLK3* SNP pairs may have an impact on PCa aggressiveness by altering specific gene expression.

### Gene interaction network with *KLK3*

**Figure 5:**
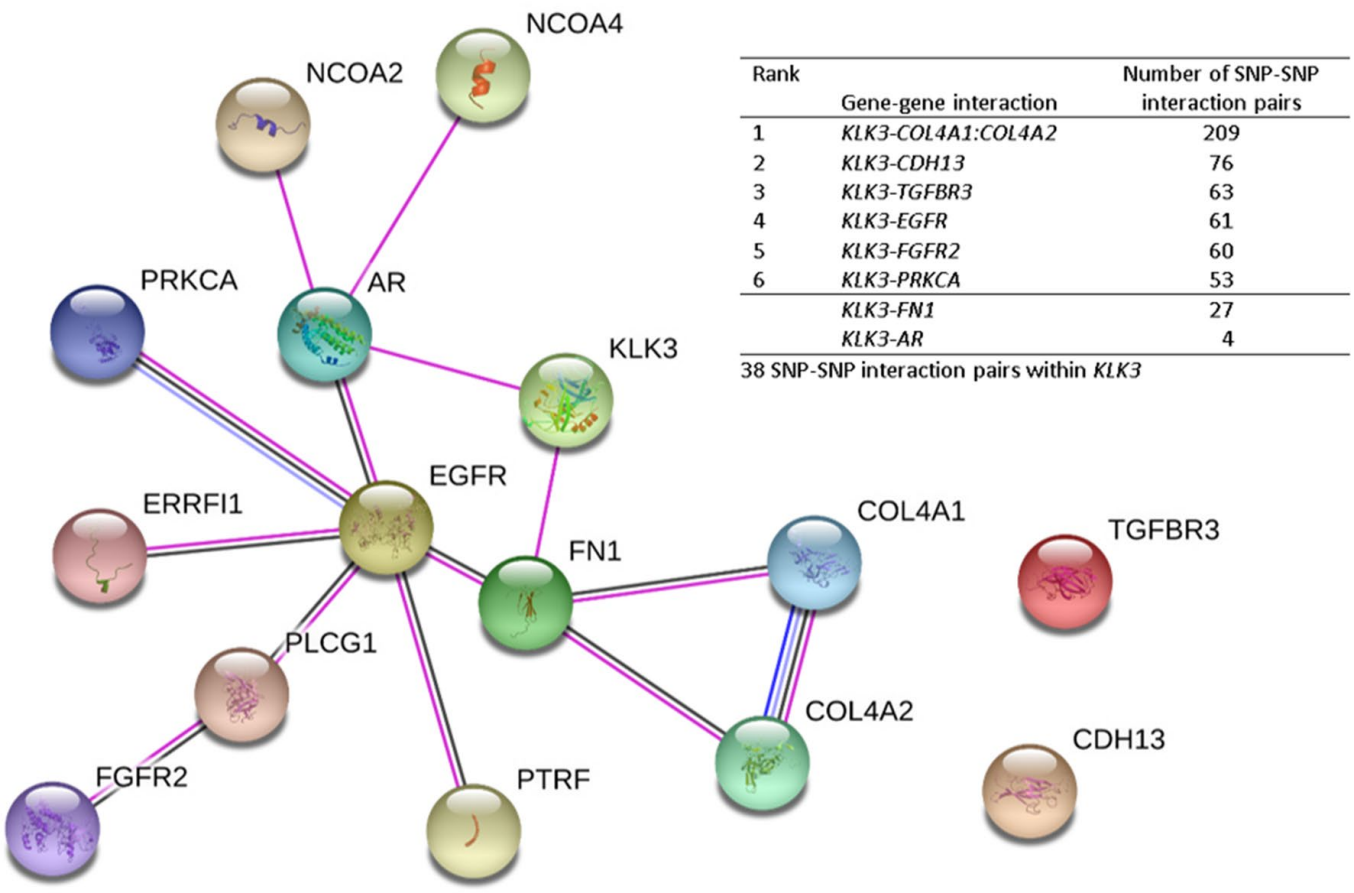
*KLK3* gene–gene interaction network based on common genes interacted with *KLK3. KLK3* and *AR* are in the androgen pathway. All other genes in the table are in the angiogenesis pathway. *SNP* single-nucleotide polymorphism. The gene–gene interaction network plot was generated using the STRING software (https://string-db.org/).

For gene-level interactions, there were 6 common genes with > 50 SNP pairs from the top 3145 SNP pairs that interacted with *KLK3* and were associated with PCa aggressiveness. As shown in Fig. 5, The 6 most common gene–gene interaction pairs were *KLK3-COL4A1: COL4A2*, *KLK3-CDH13*, *KLK3-TGFBR3*, *KLK3-EGFR*, *KLK3-FGFR2*, and *KLK3-PRKCA*. We applied the STRING database (https://string-db.org/)^21^ to analyze the gene–gene (protein–protein) interaction network. We also added 2 genes (*FN1* and the androgen receptor [*AR*]) based on our literature review. Among the top 3145 pairs, there were 27 pairs for *KLK3-FN1* and 4 pairs for *KLK3-AR*. This gene–gene interaction network supports the idea that the majority of our identified SNP–SNP interactions are directly or indirectly linked with *KLK3*. *KLK3* had a direct link with *FN1* and *AR*, which both link to *EGFR*. *EGFR* is also the hub for this gene–gene interaction network. For pathway-level analyses, the summary of the within- or between-pathway interactions for the top 3145 SNP pairs is shown in Supplementary Table S6. Among our 4 target pathways, the most common pathway-pathway interactions are androgen-angiogenesis (1543 SNP pairs) and androgen-mitochondria (1138 SNP pairs) interactions. Among the 3145 SNP pairs, 98.6% were involved with *KLK3* in the androgen pathway. This explains why the androgen pathway is the hub for pathway–pathway interactions.

## Discussion

Our study identified 3 *KLK3* SNPs (rs17632542, rs62113212, and rs2569735) and 3145 SNP interaction pairs that were associated with PCa aggressiveness. The *KLK3* SNP rs17632542, which is in a strong LD with rs62113212, had been previously identified in GWAS as being associated with several PCa-related outcomes, such as the patient’s PSA level, PCa risk, and age at PCa diagnosis and the tumor’s volume, aggressiveness, and Gleason score^22^. The SNP rs2569735 has been shown to influence miRNA functions^23^. The SNP rs2735839, which has a strong LD with rs2569735, is associated with PCa aggressiveness^24^.

The prostate is an androgen-dependent organ, and SNPs in genes involved in the androgen metabolism pathway, such as *KLK3*, have been previously implicated in PCa risk and progression^22,25^. *KLK3* is a protein-coding gene on chromosome 19q13.4, and its protein product, PSA, is a serine protease exclusively secreted by the prostate gland into the seminal fluid, where it plays an important functional role in the normal physiology of the prostate^26^. PSA is not normally secreted into the bloodstream; therefore, serum PSA levels are used for screening, diagnosing, and prognosticating PCa^27^.

Our results were consistent with previously published literature. As shown in Supplementary Table S7, our literature review showed that the *KLK3* individual SNPs rs17632542, rs2569735, and rs1058205 were significantly associated with PCa aggressiveness and PSA level. *KLK3* was involved in 98.6% of the top 3145 SNP pairs associated with PCa aggressiveness; the 4 *KLK3* SNPs (rs17632542, rs2569735, rs1058205, and rs174776) contributed to 90.8% (2856 pairs) of the identified SNP pairs. Interestingly, the 2 most common SNPs (rs17632542 and rs2569735), which interacted with many other SNPs, also had significant individual effects. According to the quality-controlled gene–gene (protein–protein) association network (Fig. 5), the majority of our identified genes had a direct or indirect link with *KLK3.* In this network, *FN1* and *AR* had a direct link with *KLK3.* Both *FN1* and *AR* are linked to *EGFR*, which is the hub for this gene–gene interaction network. *KLK3* and *AR* are in the androgen pathway, and *FN1* and *EGFR* are in the angiogenesis pathway.

*FN1* is known as a ubiquitous multifunctional glycoprotein and is involved in cell growth, migration, and differentiation^28^. An 8-gene panel has reported *FN1* to distinguish high-grade PCa from indolent PCa, with a sensitivity of 93% and specificity of 70%^29^. *AR* is known to play a vital role in PCa development and progression, and PCa cells rely on androgens for proliferation and survival^30^. *KLK3* gene expression is regulated by *AR* through androgen response elements in the promoter of PSA. Both *FN1* and *AR* are regulated by miRNA-1207-3p in *PVT1*, and these two genes are overexpressed in human PCa cell lines and tissues and are associated with PCa aggressiveness^31^. We also reported that miR-3162-5p is associated with an rs1058205-T allele in *KLK3* and can regulate *KLK3* and *AR* expression^32^. The bioinformatics analyses using STRING and GeneMANIA^33^ software show the links between *KLK3*, *FN1,* and *AR*.

As shown in Fig. 5, the 6 most common genes/regions interacting with *KLK3* are all in the angiogenesis pathway. *KLK3* expression may decrease PCa aggressiveness by inhibiting angiogenesis^34^. We previously reported that several angiogenesis genes influenced PCa aggressiveness^35^. Both *COL4A1* and *COL4A2* influence angiogenesis and tumor growth. *COL4A1* was associated with cell invasion and movement as a gene in the epithelial-to-mesenchymal (EMT) transition process, and *COL4A1* gene expression has been associated with the Gleason score^36^. *COL4A2* provides a structural component for basement membranes, is known as an inhibitor of angiogenesis, and has been considered as a biomarker for screening for benign prostatic hyperplasia^37^.

*CDH13* is located on chromosome 16q24 and is a well-known tumor suppressor gene involved in cell–cell adhesion. The expression of *CDH13* has been associated with poor PCa prognosis and low proliferation rates of prostate tumor cells^38^. *TGFBR3* is one of TGF-β receptors and is abundantly expressed in PCa cells. *TGFBR3* is downregulated during the transition from benign to malignant and metastatic prostate tissues, especially in bone metastases^39^. Several studies consistently report downregulation of *TGFBR3* in prostate tumors, suggesting that it has an important role as a tumor suppressor gene^40,41^.

*KLK3* can attenuate the response to FGF2, an angiogenesis-stimulating factor that binds to *FGFR2* and reduces endothelial cell proliferation and migration, indicating *FGF2* suppressive effect during metastasis^42^. *PRKCA* is a key negative regulator of the TGF-β pathway and is downregulated in PCa tumors. Cell migration, invasion, and bone metastasis are suppressed by overexpression of *PRKCA*. Further, expression of *PRKCA* has been correlated with clinical variables, such as PSA levels, Gleason score, and metastasis status of patients with PCa^39^.

For the non-*KLK3* SNP pairs, *KPNA3,* with 21 SNP pairs, was the most common gene, and there were 5 pairs for *KPNA3-AR* interaction. *KPNA3* is one of the subunits of the nuclear pore complex and plays a role in nuclear protein import^43^. In addition, the expression of *KPNA3* was inversely associated with survival. Baker et al. demonstrated that *KPNA3* knockdown inhibited cell proliferation, migration, and invasion. *KPNA3* may regulate protein transfer to promote colorectal cancer growth, metastasis, and relapse^43^.

We identified one additional SNP pair (rs390993 in *RIPK2* + rs473640 in *NOS1*) using the AA_Full model. The receptor-interacting serine/threonine-protein kinase 2 (RIPK2) was one of the key regulators of the immune response^44^. NO synthases (NOS) generate nitric oxide (NO), which can regulate tumorigenesis. Increased nitric oxide level by NOS is cytotoxic to cancer cells^45^. NOS1 downregulation reduced the growth of chemokine expressing fibroblasts and their ability to promote tumor formation in prostate cancer cells^46^. Although these genes' role has not been studied in PCa aggressiveness, *RIPK2* polymorphisms were associated with risk of gastric^47^ and breast^48^ cancers. *NOS1* polymorphisms were associated with the risk of various cancers, such as pancreatic^49^, glioma^50^, colorectal cancer, and melanoma^51^.

Our validated SNP–SNP interactions have been supported by both the protein–protein interaction network and eQTL results. As shown in Supplementary Table S5 and Supplementary Fig. S6, SNP interactions between *CAVIN1* and *KLK3* influenced *SRD5A2* expression. *CAVIN1* has been shown to reduce lymphangiogenesis and angiogenesis^52^. As shown in Fig. 5, *CAVIN1* links to *KLK3* through *EGFR* and *AR* according to the protein–protein interaction network. SRD5A2 converts testosterone to dihydrotestosterone, which activates ARs^53,54^. *SRD5A2* expression was significantly higher among patients with high-grade PCa vs. those with low-grade PCa^55,56^. In our previous study, we reported that the validated SNP–SNP interaction pairs of *MMP16-EGFR, MMP16-ROBO1, and MMP16-CSF1* were significantly associated with PCa aggressiveness and that *EGFR* is the hub of these interactions^13^. We observed that *MMP16, EGFR, ROBO1,* and *CSF1* interacted with *KLK3* and were associated with PCa aggressiveness. Among the top 3145 SNP pairs, 62 pairs were *KLK3-EGFR* interactions, 9 pairs were *KLK3-MMP16* interactions, 9 pairs were *KLK3-ROBO1* interactions, and 1 pair was a *KLK3-CSF1* interaction. Both proteins form *MMP16* and *EGFR* have been implicated in PCa. Several cancers that involve *EGFR* signaling often show an abnormally high expression of *MMP16*^57^. Although 2 genes (*CDH13* and *TGFBR3)* do not have a link to *KLK3* according to the protein–protein interaction network (Fig. 5), several miRNAs (such as miR_6731_5p, miR_8085, and miR_3919) contribute to the links between *KLK3* and these 2 genes^58^.

Importantly, our study demonstrated that SNP–SNP interactions could explain PCa aggressiveness better than individual SNP effects focusing on genes from the 4 selected pathways. Our study also showed that the 2-stage AA9int + SIPI approach is a powerful tool for identifying and validating SNP–SNP interactions associated with a selected phenotype. The AA9int + SIPI approach is powerful because it allows genotype subgroups with similar risk profiles or small sample sizes combined. Our risk classification system based on the model with 24 validated SNP interaction pairs is promising. Using this SNP_int_-PRS (score range 0–100), the prevalence of aggressive PCa cases from our cohort was 49.8%, 21.9%, and 7.0% with top 1%, middle 50%, and bottom 1% risk profiles, respectively. We observed that SNP pairs in the same cluster tended to be highly correlated. This may explain why we identified > 9000 significant SNP pairs. By dropping the highly correlated pairs within a cluster, we effectively reduced variable dimensions for building a multi-pair prediction model. Further biological studies will be needed to distinguish driver or passenger effects for the identified SNP pairs.

For pathway-level interactions related to PCa aggressiveness, the most common interactions were involved with the androgen pathway (such as *KLK3*), especially androgen-angiogenesis (49.1%) and androgen-mitochondria pathway interactions (36.2%, Supplementary Table S6). Angiogenesis is induced by overexpression of angiogenesis-related genes, which are regulated by many factors, including elevated androgen levels^16^. A previous study reported interaction between the regulation of angiogenesis-related genes and androgen^59^. Further, clinical therapies targeting genes involved in the androgen and angiogenesis pathways suggest an interaction between these 2 pathways^17^. In addition, recent studies have reported that androgen-related genes regulate mitochondrial respiration in PCa cells^18^. Androgen treatment leads to an increase in the activity of several metabolic pathways, including mitochondrial biogenesis and activity^60^. However, androgen repression in PCa cells decreases mitochondrial activity and cell proliferation^18^.

There are some limitations to our study. First, there is no external validation for the SNP_int_-PRS, and the identified SNP–SNP interactions may be influenced by the significance of their constituent SNP individual effects. Further analyses using large-scale studies will be needed for further verification. Second, it is challenging to identify causal SNP pairs because some SNP pairs are highly correlated, especially for SNP pairs in the same cluster. The downstream gene expression analyses or laboratory experiments will be needed to identify causal SNP pairs with biological functions. Lastly, this study only evaluated SNP–SNP interactions for the selected 4 pathways, so evaluation of SNP–SNP interactions in other pathways or genes will be required to gain a thorough understanding of the complicated gene–gene interactions associated with PCa prognosis.

In summary, this study demonstrates that *KLK3* alone and interactions between KLK3 and other identified genes play an important role in PCa aggressiveness. As shown in Fig. 5, *KLK3* directly links with *FN1* and *AR*, and other genes are indirectly linked to *KLK3* through these two genes. The *KLK3* SNP–SNP interactions can explain PCa aggressiveness better than individual SNPs. The identified SNP–SNP and gene–gene interactions may provide valuable insights for identifying downstream genes that affect PCa progression. Genetic markers, such as a panel of SNPs, are excellent predictive biomarkers as they remain unchanged during patients’ lifetime. Thus, the SNP-based scores can be a useful tool used for early prediction of future PCa progression, not just early detection when PCa progression already occurs. In addition, SNPs are tissue-independent, and can be measured non-invasively, so our SNP interaction-based PRS of PCa aggressiveness may be used clinically for disease classification and treatment guidance. Further investigation of the biological functions of the identified genes and additional validation of this prediction model is needed.

## Methods

### Study population

This study included 20 270 PCa cases (22.6% of which were aggressive PCa) with European ancestry from 21 studies within the Collaborative Oncological Gene-Environment Study (COGS) in the Prostate Cancer Association Group to Investigate Cancer Associated Alterations in the Genome (PRACTICAL) Consortium. Details of the PRACTICAL Consortium study have been previously reported^20^. PCa aggressiveness was defined as Gleason score ≥ 8, PSA level > 100 ng/mL, distant disease stage at diagnosis, or PCa-related death. Ethnic groups were defined based on ~ 37,000 uncorrelated markers that passed quality control, including ~ 1000 that were selected as ancestry informative markers. Half of the cases from each study site were randomly assigned to discovery and validation sets (10,135 cases in each set).

### Selection of genes and SNPs

We evaluated 8587 SNPs from 2122 genes within 4 pathways: the angiogenesis-, mitochondria-, miRNA-, and androgen metabolism-related pathways. These pathways were selected based on our previous studies^13,35^, the literature, and PCa GWAS. These SNPs were genotyped on a custom iCOGS Illumina array (Illumina, San Diego, CA, USA) using blood DNA samples. SNPs were excluded if they had a call rate < 95%, a call rate < 99% with minor allele frequency (MAF) < 5%, MAF < 1%, or if their genotype frequencies departed from Hardy–Weinberg equilibrium at *P* < 1 × 10^−12^.

### Statistical analyses

The effects of individual SNPs and 2-way SNP–SNP interactions on the outcome of PCa aggressiveness were evaluated. The analyses were conducted separately for the discovery set and validation set. To control for population substructure, the principal component analysis was performed. All models were adjusted for the study site and the first 6 principal components of population stratification as suggested by the PRACTICAL study^20^. For evaluating individual SNP effects, we considered three different inheritance modes: dominant [D], recessive [R], and additive [A] modes, which were assigned based on the minor allele. Logistic models were applied, and the best mode with the lowest *P* value was selected for each SNP.

We assessed 2-way SNP–SNP interactions. LD among all testing SNPs was examined based on r^2^. In order to avoid multicollinearity in modeling, we tested correlations of neighborhood SNPs within 100 kilobases using PLINK. SNPs with a strong LD of r^2^ > 0.8 were excluded from interaction analyses. A total of 5345 SNPs with a MAF > 0.05 and no strong LD (r^2^ < 0.8) were included for SNP–SNP interaction analyses.

For SNP–SNP interactions, we applied the conventional AA_Full approach using PLINK in the discovery, validation and combined sets. In addition, we applied a powerful 2-stage AA9int + SIPI approach to search intensively for SNP–SNP interactions associated with PCa aggressiveness. The AA9int approach, which treats all SNPs as having an additive inheritance mode (additive–additive patterns [AA_]), tested 9 interaction patterns of pairwise SNP–SNP interactions associated with an outcome^10^. The SIPI approach is an extended version of AA9int and tests 45 biologically meaningful SNP–SNP interaction patterns by considering 2 more inheritance modes (dominant and recessive)^9^. Thus, SIPI’s test patterns include additive–additive (AA_), dominant–dominant (DD_), dominant–recessive (DR_), recessive–dominant (RD_), and recessive–recessive (RR_) models. The AA9int and SIPI models are composed of the full-interaction model (both main effects plus interaction), the models with 1 main effect and 1 interaction, and models with interaction only. The corresponding patterns’ names are *Full*, *M1_int*, *M2_int*, or *Int_*, which represent various types of interactions with 3, 2, 2, and 1 term(s) in the statistical model equation, respectively (see the equations below). For easy interpretation, these SIPI interaction patterns can be shown as the 3 × 3 table with a heat table format using the plot3by3 function in the SIPI R package^9,10^.

$$ {\mathbf{Full}}:{\text{ SNP1 }} + {\text{ SNP2 }} + {\text{ SNP1 }} \times {\text{ SNP2}} $$

$$ {\mathbf{M1}}\_{\mathbf{int}}:{\text{ SNP1 }} + {\text{ SNP1 }} \times {\text{ SNP2}} $$

$$ {\mathbf{M2}}\_{\mathbf{int}}:{\text{ SNP2 }} + {\text{ SNP1 }} \times {\text{ SNP2}} $$

$$ {\mathbf{Int}}\_:{\text{ SNP1 }} \times {\text{ SNP2}} $$

The SNP pairs with binary modes (such as DD and RR) categorize the subjects into 2, 3, and 4 distinct risk groups for interaction-only, main + interaction, and full-interaction models, respectively. For AA models, the coding for an SNP with the additive mode is 0, 1, or 2 for the number of the target alleles, so the coding of the interaction term of 2 additive SNPs (SNP1 × SNP2) is 0, 1, 2, or 4. The details of these 2 methods were described previously^9,10^. We considered the original (0, 1, and 2 for the number of minor alleles) and reverse coding directions. For the binary outcome of PCa aggressiveness, the logistic-based AA9int was applied, and the best model was selected based on the lowest BIC value for each SNP pair. For a large-scale study, the 2-stage AA9int + SIPI approach is suggested. For performing AA9int and SIPI analyses, we used the ‘parAA9int’ and ‘parSIPI’ functions in the SIPI R package in this study. There are four related functions in the SIPI R package: SIPI, parSIPI, AA9int, and parAA9int. The parSIPI and parAA9int functions are parallel computing versions of SIPI and AA9int, which can be used for large-scale studies. We applied logistic-based AA9int and SIPI. The details of SIPI and AA9int parameter settings are listed in the SIPI R package manual. The SIPI R package and its manual are freely available for download at GitHub (https://github.com/LinHuiyi/SIPI).

As shown in Fig. 1, the 2-stage AA9int + SIPI approach was applied in the discovery set to evaluate 1.4 × 10^7^ candidate SNP pairs associated with PCa aggressiveness. Promising SNP pairs with *P* < 0.001 in the screening stage were further evaluated using the SIPI approach. The SNP pairs in the discovery set with *P* < 0.001 were further validated in the validation set. We defined significant results using *P* < 0.001 in both the discovery and validation sets and a *P* value less than the significance level of Bonferroni correction (*P* < 5.8 × 10^–6^ [= 0.05/8587 SNPs] for the individual SNP effects and *P* < 3.5 × 10^–9^ [= 0.05/1.4 × 10^7^ pairs] for interactions in the combined set). As it is well-known that Bonferroni correction is too stringent, we also used a *P* < 1 × 10^–5^ as the cutoff to select promising SNP–SNP interaction pairs. For testing whether an SNP interaction pair performed better than their composite individual SNP effects, we also compared the *P*-value of an SNP pair with *P* values of their composite individual SNP effects for SNP pairs with an interaction-only pattern. For SNP pairs with > 1 terms, the stepwise selection within a pair was applied. The methods used for SNP_int_-PRS, comparison of model performance, eQTL analyses, and the gene–gene interaction networks are listed in Supplementary Methods. All analyses were based on 2-sided tests.

## Supplementary Information

Supplementary Information.
