# Supplementary material for: KLK3 SNP–SNP interactions for prediction of prostate cancer aggressiveness
Source: Sci Rep. 2021 Apr 29;11:9264. doi: 10.1038/s41598-021-85169-7 (PMC8084951; doi:10.1038/s41598-021-85169-7)
Supplement: Supplementary file 1 — Supplementary Information. [file 41598_2021_85169_MOESM1_ESM.docx]

***KLK3* SNP-SNP interactions for prediction of prostate cancer aggressiveness**

Hui-Yi Lin^1^*, Po-Yu Huang^2^, Chia-Ho Cheng^3^, Heng-Yuan Tung^1^, Zhide Fang^1^, Anders E. Berglund^3^, Ann Chen^3^, Jennifer French-Kwawu^1^, Darian Harris^1^, Julio Pow-Sang^4^, Kosj Yamoah^5^, John L. Cleveland^6^, Shivanshu Awasthi^7^, Robert J. Rounbehler^6^, Travis Gerke^7^, Jasreman Dhillon^8^, Rosalind Eeles^9,10^, Zsofia Kote-Jarai^9^, Kenneth Muir^11,12^, UKGPCS collaborators*, Johanna Schleutker^13,14^, Nora Pashayan^15,16,17^, APCB (Australian Prostate Cancer BioResource)^*^, David E. Neal ^18,19^, Sune F. Nielsen^20,21^, Børge G. Nordestgaard^20,21^, Henrik Gronberg^22^, Fredrik Wiklund^22^, Graham G. Giles^23,24,25^, Christopher A. Haiman^26^, Ruth C. Travis^27^, Janet L. Stanford^28,29^, Adam S. Kibel^30^, Cezary Cybulski^31^, Kay-Tee Khaw^32^, Christiane Maier^33^, Stephen N. Thibodeau^34^, Manuel R. Teixeira^35,36^, Lisa Cannon-Albright^37,38^, Hermann Brenner^39,40,41^, Radka Kaneva^42^, Hardev Pandha^43^, the PRACTICAL consortium^*^, Srilakshmi Srinivasan^44,45^, Judith Clements^44,45^, Jyotsna Batra^44,45^, Jong Y. Park^7^

^1^Biostatistics Program, School of Public Health, Louisiana State University Health Sciences Center, New Orleans, LA 70112, USA. ^2^Computational Intelligence Technology Center, Industrial Technology Research Institute, Hsinchu City, Taiwan. ^3^Department of Biostatistics and Bioinformatics, Moffitt Cancer Center & Research Institute, Tampa, FL 33612, USA. ^4^Department of Genitourinary Oncology, Moffitt Cancer Center & Research Institute, Tampa, FL 33612, USA. ^5^Department of Radiation Oncology, Moffitt Cancer Center & Research Institute, Tampa, FL 33612, USA. ^6^Department of Tumor Biology, Moffitt Cancer Center & Research Institute, Tampa, FL 33612, USA. ^7^Department of Cancer Epidemiology, Moffitt Cancer Center & Research Institute, Tampa, FL 33612, USA. ^8^Department of Pathology, Moffitt Cancer Center & Research Institute, Tampa, FL 33612, USA. ^9^The Institute of Cancer Research, London, SM2 5NG, UK. ^10^Royal Marsden NHS Foundation Trust, London, SW3 6JJ, UK. ^11^Division of Population Health, Health Services Research, and Primary Care, University of Manchester, Oxford Road, Manchester, M139PT, UK. ^12^Warwick Medical School, University of Warwick, Coventry, UK. ^13^Institute of Biomedicine, Kiinamyllynkatu 10, FI-20014 University of Turku, Finland. ^14^Department of Medical Genetics, Genomics, Laboratory Division, Turku University Hospital, PO Box 52, 20521 Turku, Finland. ^15^University College London, Department of Applied Health Research, London, UK. ^16^Centre for Cancer Genetic Epidemiology, Department of Oncology, University of Cambridge, Strangeways Laboratory, Worts Causeway, Cambridge, CB1 8RN, UK.^17^Department of Applied Health Research, University College London, London, WC1E 7HB, UK. ^18^Nuffield Department of Surgical Sciences, University of Oxford, Room 6603, Level 6, John Radcliffe Hospital, Headley Way, Headington, Oxford, OX3 9DU, UK. ^19^University of Cambridge, Department of Oncology, Box 279, Addenbrooke's Hospital, Hills Road, Cambridge CB2 0QQ, UK. ^20^Health and Medical Sciences, University of Copenhagen, 2200 Copenhagen, Denmark. ^21^Department of Clinical Biochemistry, Herlev and Gentofte Hospital, Copenhagen University Hospital, Herlev, 2200 Copenhagen, Denmark. ^22^Department of Medical Epidemiology and Biostatistics, Karolinska Institute, Stockholm, Sweden. ^23^Cancer Epidemiology Division, Cancer Council Victoria, 615 St Kilda Road, Melbourne, VIC 3004, Australia. ^24^ Centre for Epidemiology and Biostatistics, Melbourne School of Population and Global Health, The University of Melbourne, Grattan Street, Parkville, VIC 3010, Australia. ^25^Precision Medicine, School of Clinical Sciences at Monash Health. Monash University. Clayton, Victoria, Australia, 3168. ^26^Center for Genetic Epidemiology, Department of Preventive Medicine, Keck School of Medicine, University of Southern California/Norris Comprehensive Cancer Center, Los Angeles, CA 90015, USA. ^27^Cancer Epidemiology Unit, Nuffield Department of Population Health, University of Oxford, Oxford, OX3 7LF, UK. ^28^Division of Public Health Sciences, Fred Hutchinson Cancer Research Center, Seattle, Washington, 98109-1024, USA. ^29^Department of Epidemiology, School of Public Health, University of Washington, Seattle, Washington 98195, USA. ^30^Division of Urologic Surgery, Brigham and Womens Hospital, 75 Francis Street, Boston, MA 02115, USA. ^31^International Hereditary Cancer Center, Department of Genetics and Pathology, Pomeranian Medical University, Szczecin, Poland. ^32^Clinical Gerontology Unit, University of Cambridge, Cambridge, CB2 2QQ, UK. ^33^Humangenetik Tuebingen, Paul-Ehrlich-Str 23, D-72076 Tuebingen, Germany. ^34^Department of Laboratory Medicine and Pathology, Mayo Clinic, Rochester, MN 55905, USA. ^35^Department of Genetics, Portuguese Oncology Institute of Porto (IPO-Porto), Porto, Portugal. ^36^Biomedical Sciences Institute (ICBAS), University of Porto, Porto, Portugal. ^37^Division of Epidemiology, Department of Internal Medicine, University of Utah School of Medicine, Salt Lake City, Utah, USA. ^38^George E. Wahlen Department of Veterans Affairs Medical Center, Salt Lake City, Utah 84148, USA. ^39^Division of Clinical Epidemiology and Aging Research, German Cancer Research Center (DKFZ), D-69120, Heidelberg, Germany. ^40^German Cancer Consortium (DKTK), German Cancer Research Center (DKFZ), D-69120 Heidelberg, Germany. ^41^Division of Preventive Oncology, German Cancer Research Center (DKFZ) and National Center for Tumor Diseases (NCT), Im Neuenheimer Feld 460, 69120 Heidelberg, Germany. ^42^Molecular Medicine Center, Department of Medical Chemistry and Biochemistry, Medical University of Sofia, Sofia, 2 Zdrave Str., 1431 Sofia, Bulgaria. ^43^University of Surrey, Guildford, Surrey, GU2 7XH, UK. ^44^Translational Research Institute, Brisbane, Queensland 4102, Australia. ^45^Australian Prostate Cancer Research Centre-Qld, Institute of Health and Biomedical Innovation and School of Biomedical Sciences, Queensland University of Technology, Brisbane QLD 4059, Australia. * Lists of authors and their affiliations appear at the end of the paper.

*Correspondence: hlin1@lsuhsc.edu

**The PRACTICAL CONSORTIUM (in addition to those named in the author list)**

Information of the consortium can be found at <http://practical.icr.ac.uk/>

**Supplementary Author List**

Artitaya Lophatananon^46^, Teuvo L. J. Tammela^47^, Csilla Sipeky^13^, Anssi Auvinen^48^, Alison M. Dunning^16^, Suzanne Chambers^49,50^, Lisa Horvath^51,52^, Leire Moya^44,45^, Gail P. Risbridger^53,54^, Wayne Tilley^55^, Jenny L. Donovan^56^, Freddie C. Hamdy^57,58^, Richard M. Martin^59,60,61^, Stig E. Bojesen^20,21^, Peter Iversen^62^, Martin Andreas Røder^62^, Melissa C. Southey^25^, Robert J. MacInnis^23,24^, Fredrick R. Schumacher^63,64^, Loic Le Marchand^65^, Xin Sheng^26^, Tim J. Key^27^, Elaine A. Ostrander^66^, Milan S. Geybels^28^, Bettina F. Drake^67^, Robert Szulkin^68,69^, Markus Aly^22,70,71^, Dominika Wokolorczyk^31^, Jan Lubinski^31^, Thomas A. Sellers^7^, Manuel Luedeke^33^, Thomas Schnoeller^72^, Thomas Schnoeller^72^, Shannon K. McDonnell^73^, Daniel J. Schaid^73^, Paula Paulo^35,74^, Andreia Brandão^35,74^, Craig C Teerlink^37,38^, Xin Gao^39^, Bernd Holleczek^75^, Ben Schöttker^39^, Chavdar Slavov^76^, Vanio Mitev^42^, Agnieszka Michael^43^

^46^Division of Population Health, Health Services Research and Primary Care, School of Health Sciences, Faculty of Biology, Medicine and Health, University of Manchester, Manchester, UK, M139PT. ^47^Department of Urology, Tampere University Hospital, Tampere, Finland. ^48^Unit of Health Sciences, Faculty of Social Sciences, Tampere University, Tampere, Finland. ^49^University of Technology, Sydney. ^50^Cancer Council Queensland, Fortitude Valley, QLD 4006, Australia.^51^Chris O'Brien Lifehouse (COBLH), Camperdown, Sydney, NSW 2010, Australia. ^52^Garvan Institute of Medical Research, Sydney NSW 2010, Australia. ^53^Department of Anatomy and Developmental Biology, Biomedicine Discovery Institute, Monash University, Melbourne, Victoria 3800, Australia. ^54^Prostate Cancer Translational Research Program, Cancer Research Division, Peter MacCallum Cancer Centre, Melbourne, VIC 3000, Australia.^55^Dame Roma Mitchell Cancer Research Laboratories, University of Adelaide, Adelaide, South Australia, Australia.^56^Population Health Sciences, Bristol Medical School, University of Bristol, BS8 2PS, UK. ^57^Nuffield Department of Surgical Sciences, University of Oxford, Oxford, OX1 2JD, UK. ^58^Faculty of Medical Science, University of Oxford, John Radcliffe Hospital, Oxford, UK.  ^59^Population Health Sciences, Bristol Medical School, University of Bristol, BS8 2PS, UK. ^60^National Institute for Health Research (NIHR) Biomedical Research Centre, University of Bristol, Bristol, BS8 1TH, UK. ^61^Medical Research Council (MRC) Integrative Epidemiology Unit, University of Bristol, Bristol, BS8 2BN, UK. ^62^Copenhagen Prostate Cancer Center, Department of Urology, Rigshospitalet, Copenhagen University Hospital, DK-2730 Herlev, Denmark. ^63^Department of Population and Quantitative Health Sciences, Case Western Reserve University, Cleveland, OH 44106-7219, USA. ^64^Seidman Cancer Center, University Hospitals, Cleveland, OH 44106, USA. ^65^Epidemiology Program, University of Hawaii Cancer Center, Honolulu, HI 96813, USA. ^66^National Human Genome Research Institute, National Institutes of Health, 50 South Drive, Rm. 5351, Bethesda, MD 20892, USA. ^67^Washington University School of Medicine, 660 S. Euclid Avenue, Campus Box 8242, St. Louis, MO 63110, USA. ^68^Division of Family Medicine, Department of Neurobiology, Care Science and Society, Karolinska Institutet, Huddinge, SE-171 77 Stockholm, Sweden. ^69^Scandinavian Development Services, Danderyd, 182 33, Sweden. ^70^Department of Molecular Medicine and Surgery, Karolinska Institutet, and Department of Urology, Karolinska University Hospital, Solna, 171 76 Stockholm. ^71^Department of Urology, Karolinska University Hospital, Stockholm, Sweden. ^72^Department of Urology, University Hospital Ulm, Germany. ^73^Division of Biomedical Statistics & Informatics, Mayo Clinic, Rochester, MN 55905, USA. ^74^Cancer Genetics Group, IPO-Porto Research Center (CI-IPOP), Portuguese Oncology Institute of Porto (IPO-Porto), Porto, Portugal. ^75^Saarland Cancer Registry, 66119 Saarbrücken, Germany.^76^Department of Urology and Alexandrovska University Hospital, Medical University of Sofia, 1431 Sofia, Bulgaria.

**Funding for the CRUK study and PRACTICAL consortium:** This work was supported by the Canadian Institutes of Health Research, European Commission's Seventh Framework Programme grant agreement n° 223175 (HEALTH-F2-2009-223175), Cancer Research UK Grants C5047/A7357, C1287/A10118, C1287/A16563, C5047/A3354, C5047/A10692, C16913/A6135, and The National Institute of Health (NIH) Cancer Post-Cancer GWAS initiative grant: No. 1 U19 CA 148537-01 (the GAME-ON initiative).

**COGS acknowledgment:** This study would not have been possible without the contributions of the following: Per Hall (COGS); Douglas F. Easton, Paul Pharoah, Kyriaki Michailidou, Manjeet K. Bolla, Qin Wang (BCAC), Andrew Berchuck (OCAC), Rosalind A. Eeles, Douglas F. Easton, Ali Amin Al Olama, Zsofia Kote-Jarai, Sara Benlloch (PRACTICAL), Georgia Chenevix-Trench, Antonis Antoniou, Lesley McGuffog, Fergus Couch and Ken Offit (CIMBA), Joe Dennis, Alison M. Dunning, Andrew Lee, and Ed Dicks, Craig Luccarini and the staff of the Centre for Genetic Epidemiology Laboratory, Javier Benitez, Anna Gonzalez-Neira and the staff of the CNIO genotyping unit, Jacques Simard and Daniel C. Tessier, Francois Bacot, Daniel Vincent, Sylvie LaBoissière and Frederic Robidoux and the staff of the McGill University and Génome Québec Innovation Centre, Stig E. Bojesen, Sune F. Nielsen, Borge G. Nordestgaard, and the staff of the Copenhagen DNA laboratory, and Julie M. Cunningham, Sharon A. Windebank, Christopher A. Hilker, Jeffrey Meyer and the staff of Mayo Clinic Genotyping Core Facility

Funding for the iCOGS infrastructure came from: the European Community's Seventh Framework Programme under grant agreement n° 223175 (HEALTH-F2-2009-223175) (COGS), Cancer Research UK (C1287/A10118, C1287/A 10710, C12292/A11174, C1281/A12014, C5047/A8384, C5047/A15007, C5047/A10692, C8197/A16565), the National Institutes of Health (CA128978) and Post-Cancer GWAS initiative (1U19 CA148537, 1U19 CA148065 and 1U19 CA148112 - the GAME-ON initiative), the Department of Defence (W81XWH-10-1-0341), the Canadian Institutes of Health Research (CIHR) for the CIHR Team in Familial Risks of Breast Cancer, Komen Foundation for the Cure, the Breast Cancer Research Foundation, and the Ovarian Cancer Research Fund.

**Additional funding and acknowledgments from studies in PRACTICAL:**

CAPS / STHM1

The Department of Medical Epidemiology and Biostatistics, Karolinska Institute, Stockholm, Sweden was supported by the Cancer Risk Prediction Center (CRisP; www.crispcenter.org), a Linneus Centre (Contract ID 70867902) financed by the Swedish Research Council, Swedish Research Council (grant no K2010-70X-20430-04-3), the Swedish Cancer Foundation (grant no 09-0677), the Hedlund Foundation, the Soederberg Foundation, the Enqvist Foundation, ALF funds from the Stockholm County Council. Stiftelsen Johanna Hagstrand och Sigfrid Linner's Minne, Karlsson's Fund for urological and surgical research.

We thank and acknowledge all of the participants in the Stockholm-1 study. We thank Carin Cavalli-Bjoerkman and Ami Roennberg Karlsson for their dedicated work in the collection of data. Michael Broms is acknowledged for his skillful work with the databases. KI Biobank is acknowledged for handling the samples and for DNA extraction. Hans Wallinder at Aleris Medilab and Sven Gustafsson at Karolinska University Laboratory are thanked for their good cooperation in providing historical laboratory results.

CPCS1 / CPCS2

Department of Clinical Biochemistry, Herlev and Gentofte Hospital, Copenhagen University Hospital, Herlev Ringvej 75, DK-2730 Herlev, Denmark.

We thank participants and staff of the Copenhagen General Population Study for their important contributions.

EPIC

The coordination of EPIC was financially supported by the European Commission (DG-SANCO) and the International Agency for Research on Cancer. The national cohorts (that recruited male participants) are supported by Danish Cancer Society (Denmark); German Cancer Aid, German Cancer Research Center (DKFZ), Federal Ministry of Education and Research (BMBF), Deutsche Krebshilfe, Deutsches Krebsforschungszentrum and Federal Ministry of Education and Research (Germany); the Hellenic Health Foundation (Greece); Associazione Italiana per la Ricerca sul Cancro-AIRC-Italy and National Research Council (Italy); Dutch Ministry of Public Health, Welfare and Sports (VWS), Netherlands Cancer Registry (NKR), LK Research Funds, Dutch Prevention Funds, Dutch ZON (Zorg Onderzoek Nederland), World Cancer Research Fund (WCRF), Statistics Netherlands (The Netherlands); Health Research Fund (FIS), PI13/00061 to Granada; , PI13/01162 to EPIC-Murcia), Regional Governments of Andalucía, Asturias, Basque Country, Murcia and Navarra, ISCIII RETIC (RD06/0020) (Spain); Swedish Cancer Society, Swedish Research Council and County Councils of Skåne and Västerbotten (Sweden); Cancer Research UK (14136 to EPIC-Norfolk;

C570/A16491 and C8221/A19170 to EPIC-Oxford), Medical Research Council

(1000143 to EPIC-Norfolk, MR/M012190/1 to EPIC-Oxford) (United Kingdom). For information on how to submit an application for gaining access to EPIC data and/or biospecimens, please follow the instructions at http://epic.iarc.fr/access/index.php

ESTHER

The ESTHER study was supported by a grant from the Baden Württemberg Ministry of Science, Research and Arts. The ESTHER group would like to thank Hartwig Ziegler, Sonja Wolf, Volker Hermann, Heiko Müller, Karina Dieffenbach, Katja Butterbach for valuable contributions to the study.

FHCRC

The FHCRC studies were supported by grants R01-CA056678, R01-CA082664, and R01-CA092579 from the US National Cancer Institute, National Institutes of Health, with additional support from the Fred Hutchinson Cancer Research Center. We thank all the men who participated in these studies.

IPO-Porto

The IPO-Porto study was funded by Fundação para a Ciência e a Tecnologia (FCT; UID/DTP/00776/2013 and PTDC/DTP-PIC/1308/2014) and by IPO-Porto Research Center (CI-IPOP-16-2012 and CI-IPOP-24-2015). MC and MPS are research fellows from Liga Portuguesa Contra o Cancro, Núcleo Regional do Norte. SM is a research fellow from FCT (SFRH/BD/71397/2010). We would like to express our gratitude to all patients and families who have participated in this study.

MAYO

The Mayo group was supported by the US National Cancer Institute (R01CA72818).

MCCS

The Melbourne Collaborative Cohort Study (MCCS) cohort recruitment was funded by VicHealth and Cancer Council Victoria. The MCCS was further supported by Australian National Health and Medical Research Council grants 209057 and 396414 and by infrastructure provided by Cancer Council Victoria. Cases and their vital status were ascertained through the Victorian Cancer Registry and the Australian Institute of Health and Welfare, including the National Death Index and the Australian Cancer Database.

MEC

The MEC was supported by NIH grants CA63464, CA54281, CA098758, and CA164973.

MOFFITT

The Moffitt group was supported by the US National Cancer Institute (R01CA128813, PI: J.Y. Park).

PCMUS

The PCMUS study was supported by the Bulgarian National Science Fund, Ministry of Education and Science (contract DOO-119/2009; DUNK01/2-2009; DFNI-B01/28/2012) with additional support from the Science Fund of Medical University - Sofia (contract 51/2009; 8I/2009; 28/2010).

ProtecT

ProtecT would like to acknowledge the support of The University of Cambridge, Cancer Research UK. Cancer Research UK grants (C8197/A10123) and (C8197/A10865) supported the genotyping team. We would also like to acknowledge the support of the National Institute for Health Research which funds the Cambridge Bio-medical Research Centre, Cambridge, UK. We would also like to acknowledge the support of the National Cancer Research Prostate Cancer: Mechanisms of Progression and Treatment (PROMPT) collaborative (grant code G0500966/75466) which has funded tissue and urine collections in Cambridge. We are grateful to the staff at the Welcome Trust Clinical Research Facility, Addenbrooke’s Clinical Research Centre, Cambridge, UK for their help in conducting the ProtecT study. We also acknowledge the support of the NIHR Cambridge Biomedical Research Centre, the DOH HTA (ProtecT grant) and the NCRI / MRC (ProMPT grant) for help with the bio-repository. The UK Department of Health funded the ProtecT study through the NIHR Health Technology Assessment Programme (projects 96/20/06, 96/20/99). The ProtecT trial and its linked ProMPT and CAP (Comparison Arm for ProtecT) studies are supported by Department of Health, England; Cancer Research UK grant number C522/A8649, Medical Research Council of England grant number G0500966, ID 75466 and The NCRI, UK. The epidemiological data for ProtecT were generated though funding from the Southwest National Health Service Research and Development. DNA extraction in ProtecT was supported by USA Dept of Defense award W81XWH-04-1-0280, Yorkshire Cancer Research, and Cancer Research UK. The authors would like to acknowledge the contribution of all members of the ProtecT study research group. The views and opinions expressed therein are those of the authors and do not necessarily reflect those of the Department of Health of England. The bio-repository from ProtecT is supported by the NCRI (ProMPT) Prostate Cancer Collaborative and the Cambridge BMRC grant from NIHR. We acknowledge support from the National Cancer Research Institute (National Institute of Health Research (NIHR) Collaborative Study: “Prostate Cancer: Mechanisms of Progression and Treatment (PROMPT)” (grant G0500966/75466). We thank the National Institute for Health Research, Hutchison Whampoa Limited, the Human Research Tissue Bank (Addenbrooke’s Hospital), and Cancer Research UK. The authors would like to thank those men with prostate cancer and the subjects who have donated their time and their samples to the Cambridge Biorepository, which were used in this research. We also would like to acknowledge to support of the research staff in S4 who so carefully curated the samples and the follow-up data (Jo Burge, Marie Corcoran, Anne George, and Sara Stearn).

QLD

The QLD research is supported by The National Health and Medical Research Council (NHMRC) Australia Project Grants [390130, 1009458] and NHMRC Career Development Fellowship, Cancer Australia PdCCRS and Cancer Council Queensland funding to J Batra. The QLD team would like to acknowledge and sincerely thank the urologists, pathologists, data managers and patient participants who have generously and altruistically supported the QLD cohort.

SEARCH

SEARCH is funded by a program grant from Cancer Research UK [C490/A10124] and supported by the UK National Institute for Health Research Biomedical Research Centre at the University of Cambridge. The University of Cambridge has received salary support with respect to PP from the NHS in the East of England through the Clinical Academic Reserve.

TAMPERE

The Tampere (Finland) study was supported by the Academy of Finland (251074), The Finnish Cancer Organisations, Sigrid Juselius Foundation, and the Competitive Research Funding of the Tampere University Hospital (X51003). The PSA screening samples were collected by the Finnish part of ERSPC (European Study of Screening for Prostate Cancer). TAMPERE would like to thank Riina Liikanen, Liisa Maeaettaenen and Kirsi Talala for their work on samples and databases.

UKGPCS

UKGPCS would also like to thank the following for funding support: The Institute of Cancer Research and The Everyman Campaign, The Prostate Cancer Research Foundation, Prostate Research Campaign UK (now Prostate Action), The Orchid Cancer Appeal, The National Cancer Research Network UK, The National Cancer Research Institute (NCRI) UK. We are grateful for the support of NIHR funding to the NIHR Biomedical Research Centre at The Institute of Cancer Research and The Royal Marsden NHS Foundation Trust. UKGPCS should also like to acknowledge the NCRN nurses, data managers and Consultants for their work in the UKGPCS study. UKGPCS would like to thank all urologists and other persons involved in the planning, coordination, and data collection of the study. KM and AL were in part supported by the NIHR Manchester Biomedical Research Centre.

ULM

The Ulm group received funds from the German Cancer Aid (Deutsche Krebshilfe).

UTAH

The Keith and Susan Warshaw Fund, C. S. Watkins Urologic Cancer Fund and The Tennity Family Fund supported the Utah study. The project was supported by Award Number P30CA042014 from the National Cancer Institute

WUGS

WUGS would like to thank the following for funding support: The Anthony DeNovi Fund, the Donald C. McGraw Foundation, and the St. Louis Men’s Group Against Cancer.

**Supplementary Methods**

Development of SNP-interaction polygenic risk score (SNP_int_ -PRS)

For building a prediction model of PCa aggressiveness by considering SNP-SNP interactions, the candidate SNP pairs were selected based on the combined set, which had a large sample size for better reliability than the subset. We dropped 82 SNP pairs with >200 missing values, and a total of 3,062 (=3144-82) SNP pairs were candidates for model building. Because many of the top SNP pairs shared a hub SNP, an “SNP cluster” was defined as a group of SNP pairs involved with one common SNP (such as SNP_A_-SNP_B_, SNP_A_-SNP_C_ and SNP_A_-SNP_D_ are in the A cluster). We observed that the SNP pairs in the same cluster tended to be correlated with each other. For variable reduction and avoiding multicollinearity issue in model building, the following steps were applied:

1. Within each cluster, correlations between SNP pairs with an interaction-only pattern with an additive mode (such as AA_int_oo) were tested using the Pearson correlation (r) for the interaction-only model. For the interaction-only model with binary dominant or recessive inheritance modes (such as DD_int_oo and DR_int_oo), the Phi correlation (φ), which measures the association between two binary variables, was applied.
2. For each SNP cluster, the set of low-correlated pairs (r or φ< 0.7) and the pairs with a complicated interaction pattern (such as AA_M1_int_o1) associated with PCa aggressiveness were tested using stepwise selection with a significance level of 0.1. The candidate SNP pairs for model building are pairs selected within the clusters and other pairs, which were not in the clusters.

Using these selected candidate SNP pairs, the stepwise selection with a p<0.01 and p<1x10^-5^ were applied for building two multi-pair models in the combined set. The SNP-interaction polygenic risk score (SNP_int_ -PRS) of PCa aggressiveness was developed based on the predicted probabilities of the logistic model with the selected SNP interaction pairs and without other covariates. The calculation of this SNP_int_ –PRS is listed below.

Raw -PRS$={Prob}_{predicted}=\frac{exp(\beta_{1}{Pair}_{1}+\beta_{2}{Pair}_{2}+\ldots+\beta_{k}{Pair}_{k})}{1+exp(\beta_{1}{Pair}_{1}+\beta_{2}{Pair}_{2}+\ldots+\beta_{k}{Pair}_{k})}$

SNP_int_ -PRS = (Raw-PRS – a minimum of Raw-PRS)*100/ (range of Raw-PRS)

where $\beta_{k}$ is the log odds ratio for the SNP pair_k_ in the logistic model of PCa aggressiveness. For easy interpretation, this PRS was rescaled to a range of 0-100. Higher scores indicate a higher risk of PCa aggressiveness. We classified the risk groups into seven sub-groups based on the PRS’s distribution (<1, 1-10, 10-25, 25-75, 75-90, 90-99, and ≥ 99%^1^.

For building a prediction model for the verified individual SNP effects, we applied the stepwise selection with a p<1x10^-5^ for the three significant *KLK3* SNPs listed in Table 1 in the combined set. For comparing the performance of these three individual-effect models and the proposed interaction model (SNP_int_ -PRS), we compared them using the area under the receiver operator characteristics curve (AUC), R^2^, and Bayesian information criterion (BIC). For internal validation of the SNPint -PRS, the 95% confidence interval of AUC based on the 1,000 bootstrap samples was calculated.

eQTL analyses

The biological functions of the identified SNP pairs were evaluated using 2-way expression quantitative trait loci (eQTLs) analyses to assess the 2-to-1 relationship between one SNP interaction pair (two SNPs) and one gene expression. We performed 4.5x10^6^ (=1459x3056) 2-way eQTL analyses using the linear-model based SIPI approach with the normalized and log2 transformed gene expression levels as outcomes. All models were adjusted for DNA methylation and copy number variations (CNV) if they were available. We used a total of 231 PCa cases of European ancestry and good pathological quality in the Cancer Genome Atlas (TCGA) PCa study. Of the 495 PCa cases with SNP and gene expression data, there were 352 Whites, defined as >80% of European ancestry based on our previous study ^2^. The samples of 231 white cases passed pathology quality control ^3^. SNP data were generated using Affymetrix Genome-Wide Human SNP Array 6.0, and mRNA expression profiling was performed using Illumina HiSeq 2000 RNA Sequencing. From the top 9,492 SNP pairs identified in the PRACTICAL data, there were 3,056 pairs available in the TCGA after excluding SNPs with >80%­­ missing and a MAF<0.05. For gene expression, the expression of the 1,459 genes in the four candidate pathways was available after excluding genes with >80% zero-counts.

Gene-gene interaction network

To investigate potential biological functions of our identified gene-gene interactions, the protein-protein interaction network by STRING database was performed^4^. We entered candidate genes based on the top 3,144 SNP pairs and a literature review. Only solid associations with experiments, co-expression, and co-occurrence with medium confidence (confidence score ≥ 0.4) were considered. The confidence score has a range of 0-1 from low to high confidence. In the network, a node represents a protein, and an edge indicates an interaction of two connected proteins.

**Reference for supplement materials:**

1. Schumacher FR*, et al.* Association analyses of more than 140,000 men identify 63 new prostate cancer susceptibility loci. *Nat Genet* **50**, 928-936 (2018).

2. Lin HY, Cheng CH, Chen DT, Chen YA, Park JY. Coexpression and expression quantitative trait loci analyses of the angiogenesis gene-gene interaction network in prostate cancer. *Transl Cancer Res* **5**, S951-S963 (2016).

3. Cancer Genome Atlas Research N. The Molecular Taxonomy of Primary Prostate Cancer. *Cell* **163**, 1011-1025 (2015).

4. Szklarczyk D*, et al.* The STRING database in 2017: quality-controlled protein-protein association networks, made broadly accessible. *Nucleic Acids Res* **45**, D362-D368 (2017).

5. Eeles RA*, et al.* Multiple newly identified loci associated with prostate cancer susceptibility. *Nature genetics* **40**, 316 (2008).

6. Gudmundsson J*, et al.* Genetic correction of PSA values using sequence variants associated with PSA levels. *Science translational medicine* **2**, 62ra92-62ra92 (2010).

7. Sun J*, et al.* Genome-wide association study identified novel genetic variant on SLC45A3 gene associated with serum levels prostate-specific antigen (PSA) in a Chinese population. *Human genetics* **132**, 423-429 (2013).

8. Lange EM*, et al.* Genome-wide association scan for variants associated with early-onset prostate cancer. *PloS one* **9**, e93436 (2014).

9. Knipe DW*, et al.* Genetic variation in prostate-specific antigen–detected prostate cancer and the effect of control selection on genetic association studies. *Cancer Epidemiology and Prevention Biomarkers* **23**, 1356-1365 (2014).

10. Terao C*, et al.* A genome-wide association study of serum levels of prostate-specific antigen in the Japanese population. *Journal of medical genetics* **51**, 530-536 (2014).

11. Hoffmann TJ*, et al.* A large multiethnic genome-wide association study of prostate cancer identifies novel risk variants and substantial ethnic differences. *Cancer discovery* **5**, 878-891 (2015).

12. Hoffmann TJ*, et al.* Genome-wide association study of prostate-specific antigen levels identifies novel loci independent of prostate cancer. *Nature communications* **8**, 1-11 (2017).

13. Choe EK*, et al.* Search for genetic factor association with cancer-free prostate-specific antigen level elevation on the basis of a genome-wide association study in the Korean population. *European Journal of Cancer Prevention* **27**, 453-460 (2018).

14. Schumacher FR*, et al.* Association analyses of more than 140,000 men identify 63 new prostate cancer susceptibility loci. *Nature genetics* **50**, 928-936 (2018).

15. Takata R*, et al.* 12 new susceptibility loci for prostate cancer identified by genome-wide association study in Japanese population. *Nature communications* **10**, 1-10 (2019).

16. Bensen JT, Xu Z, Smith GJ, Mohler JL, Fontham ET, Taylor JA. Genetic polymorphism and prostate cancer aggressiveness: A case‐only study of 1,536 GWAS and candidate SNPs in African‐Americans and European‐Americans. *The Prostate* **73**, 11-22 (2013).

17. Berndt SI*, et al.* Two susceptibility loci identified for prostate cancer aggressiveness. *Nature communications* **6**, 1-7 (2015).

Table S1. Top 17 SNP interaction pairs (p<10^-6^) involved with **non-*KLK3*** genes associated with prostate cancer aggressiveness

| **SNP1** | **SNP2** | **Interaction pattern label^a^** | **Pattern details** | **SNP1**  **Min<Maj**  **(MAF)** | **SNP2**  **Min<Maj**  **(MAF)** | **Combined**  ***P* value^b^** | **OR (95% CI)^b^** | **Dis. *P* value^b^** | **Val. *P* value^b^** | **Gene1** | **Gene2** |
| --- | --- | --- | --- | --- | --- | --- | --- | --- | --- | --- | --- |
| rs2266967 | rs7446 | DD_int_oo | (CA/AA+ GA/AA) vs. others | A<C (0.50) | A<G (0.31) | 3.6X10^-8^ | 1.21 (1.13- 1.30) | 2.8X10^-4^ | 1.2X10^-5^ | *MAPK1* | *KPNA3* |
| rs3135831 | rs7446 | DD_int_oo | (GA/AA+ GA/AA) vs. others | A<G (0.44) | A<G (0.31) | 3.7X10^-8^ | 1.22 (1.14- 1.31) | 1.9X10^-4^ | 5.2X10^-5^ | *FGFR2* | *KPNA3* |
| rs1433165 | rs7446 | DD_int_oo | (GA/AA+ GA/AA) vs. others | A<G (0.41) | A<G (0.31) | 4.9X10^-8^ | 1.22 (1.13- 1.31) | 5.7X10^-5^ | 1.8X10^-4^ | *ANGPT1* | *KPNA3* |
| rs12541789 | rs7446 | DD_int_oo | (AG/GG+ GA/AA) vs. others | G<A (0.40) | A<G (0.31) | 6.7X10^-8^ | 1.22 (1.13- 1.31) | 1.1X10^-4^ | 4.8X10^-5^ | *DEPTOR* | *KPNA3* |
| rs681309 | rs7446 | DD_int_oo | (GA/AA+ GA/AA) vs. others | A<G (0.43) | A<G (0.31) | 8.6X10^-8^ | 1.21 (1.13- 1.3) | 2.4X10^-5^ | 1.1X10^-4^ | *SLC25A45* | *KPNA3* |
| rs7317591 | rs7446 | RD_int_rr | (AA/AG+ GG) vs. others | C<A (0.49) | A<G (0.31) | 1.7X10^-7^ | 0.83 (0.77- 0.89) | 9.8X10^-5^ | 4.1X10^-4^ | *COL4A1* | *KPNA3* |
| rs1518887 | rs7326449 | DD_int_oo | (AG/GG+ AG/GG) vs. others | G<A (0.39) | G<A (0.21) | 2.1X10^-7^ | 1.23 (1.14- 1.33) | 3.2X10^-4^ | 1.7X10^-4^ | *LINC01811* | *COL4A2* |
| rs2317676 | rs7802277 | DR_M2_int_o2 | (AA+ AA)  (AG/GG+ AA) vs. others | G<A (0.07) | A<G (0.142) | 8.3x10^-4^  3.7x10^-7^ | 0.62 (0.47- 0.82)  3.13 (1.79-5.47) | 9.2x10^-4^ | 9.2x10^-4^ | *ITGB3* | *MTCYBP42* |
| rs1331981 | rs7326449 | DD_int_rr | (GG+ AA) vs. others | A<G (0.07) | G<A (0.21) | 3.8X10^-7^ | 0.84 (0.78- 0.9) | 1.8X10^-4^ | 5.4X10^-4^ | *LINC02544* | *COL4A2* |
| rs16966598 | rs1985152 | AA_int_oo | SNP1xSNP2  (C,G) | C<G (0.15) | G<A (0.37) | 3.8X10^-7^ | 0.84 (0.79- 0.9) | 2.1X10^-4^ | 2.5X10-^4^ | *NOS2* | *JAZF1* |
| rs7446 | rs4919687 | AA_int_oo | SNP1xSNP2 (A,A) | A<G (0.31) | A<G (0.29) | 5.2X10^-7^ | 1.13 (1.08- 1.18) | 1.4X10^-5^ | 7.3X10^-5^ | *KPNA3* | *CYP17A1* |
| rs1523303 | rs2266967 | AA_int_ro | SNP1xSNP2 (G,A) | A<G (0.38) | A<C (0.50) | 6.6X10^-7^ | 1.07 (1.04- 1.10) | 2.0X10^-4^ | 8.1X10^-4^ | *TGFA* | *MAPK1* |
| rs219158 | rs2361634 | DD_int_oo | (AG/GG+ AG/GG) vs. others | G<A (0.38) | G<A (0.07) | 7.1X10^-7^ | 1.48 (1.27- 1.72) | 2.5X10^-4^ | 2.1X10^-4^ | *LTBP1* | *AR* |
| rs13035938 | rs8082010 | DD_int_oo | (AG/GG+ GA/AA) vs. others | G<A (0.28) | A<G (0.07) | 7.5X10^-7^ | 0.69 (0.60- 0.80) | 2.1X10^-4^ | 4.5X10^-4^ | *TFPI* | *PRKCA* |
| rs12143015 | rs1518887 | AA_int_oo | SNP1xSNP2 (A,G) | A<G (0.22) | G<A (0.39) | 7.9X10^-7^ | 1.13 (1.08- 1.18) | 5.5X10^-4^ | 3.6X10^-4^ | *RASAL2* | *LINC01811* |
| rs1649949 | rs16958619 | DD_int_oo | (GA/AA+ CA/AA) vs. others | A<G (0.19) | A<C (0.13) | 8.7X10^-7^ | 1.35 (1.20- 1.51) | 3.7X10^-4^ | 7.3X10^-4^ | *NRG3* | *CDH13* |
| rs4148853 | rs905557 | AA_int_oo | SNP1xSNP2 (A,A) | A<G (0.19) | A<G (0.13) | 9.3X10^-7^ | 1.26 (1.15- 1.39) | 8.9X10^-4^ | 3.2X10^-4^ | *ABCB8* | *OXR1* |

^a^ SIPI pattern interpretation: The first 2 letters represent inheritance modes of the first SNP (or SNP1) and the 2^nd^ SNPs (or SNP2); mode (A, additive mode; D, dominant mode; R, recessive mode; “(SNP1 mode)(SNP2 mode)_int_(SNP1 code)(SNP2 code)” is for interaction-only pattern; “Mϕ_int_”, interaction plus one main effect of SNPϕ;

SNP code (o, original coding based on the minor allele; r, reverse coding; o2: original coding for SNP2 (SNP1 with original coding)

^b^ For verified SNP pairs with P < 1×10^-5^ in the combined set and p_interaction < p_individual; all models adjusted for study site and first six principal components

Abbreviations: CI, confidence interval; Dis, discovery set; MAF, minor allele frequency; Maj, major allele; Min, minor allele; OR, odds ratio; Val, validation set.

Table S2. Variable selection procedure for the 11 SNP clusters

| Hub SNP in the cluster | Gene | No. of  top SNP pairs^a^ | No. pairs with p_int  <p_indid | No. pairs with pattern >1 term | No. low-correlated pairs (r<0.7)^b^ | No. low-correlated pairs (r<0.7) & miss<200^b^ | No. pairs associated with PCa aggr. (stepwise p<0.1)^b^ |
| --- | --- | --- | --- | --- | --- | --- | --- |
| rs2569735 | *KLK3* | 4179 | 1132 | 3 | 23 | 23 | 14 |
| rs17632542 | *KLK3* | 3488 | 769 | 3 | 4 | 4 | 5 |
| rs1058205 | *KLK3* | 1146 | 601 | 1 | 37 | 37 | 14 |
| rs174776 | *KLK3* | 397 | 350 | 1 | 38 | 38 | 9 |
| rs2271095 | *KLK3* | 93 | 93 | 0 | 16 | 15 | 8 |
| rs266876 | *KLK3* | 66 | 66 | 0 | 28 | 27 | 5 |
| rs4802755 | *KLK3* | 63 | 63 | 0 | 25 | 25 | 9 |
| rs7446 | *KPNA3* | 27 | 27 | 0 | 17 | 17 | 4 |
| rs6998 | *KLK3* | 23 | 23 | 0 | 10 | 10 | 4 |
| rs4802754 | *KLK3* | 11 | 11 | 0 | 9 | 9 | 4 |
| rs2361634 | *AR* | 10 | 10 | 0 | 6 | 6 | 2 |
| No. of unique pairs in the 11 clusters |  | 9469 | 3121 | 8 | 209 | 207 | 76 |
| No. pairs NOT in the 11 clusters |  | 23 | 23 | 1 | NA | 20 | 20 |
| Total pairs |  | **9492** | **3144** | 9 |  | 227 | **96** |

^a^Top 9,492 SNP pairs associated with PCa aggressiveness with a p-value< 1x10^-5^. Some SNP pairs included in two clusters. p_int: p-value of interaction, p_indid: p-value of individual SNP effect.

^b^r: Pearson correlation for SNP pairs with the interaction-only pattern within the clusters; candidate pairs for within-cluster stepwise selection: low-correlated pairs + pairs with a pattern >1 term excluding pairs with ≥200 missing values

Table S3. The 24-pair model associated with prostate cancer aggressiveness in the combined set

| SNP1 | SNP2 | Interaction pattern label^a^ | Pattern details | SNP1  Min<Maj  (MAF) | SNP2  Min<Maj  (MAF) | Uni-pair p^b^ | Uni-pair  OR (95%CI) | Multi-pair p^b^ | Multi-pair  OR (95%CI) | Gene1 | Gene2 |
| --- | --- | --- | --- | --- | --- | --- | --- | --- | --- | --- | --- |
| rs13035938 | rs8082010 | DD_int_oo | (GA/GG+ AG/AA) vs. others | G<A (0.28) | A<G (0.07) | 7.5X10^-7^ | 0.69 (0.6- 0.8) | 3.8X10^-8^ | 0.66 (0.57-0.76) | TFPI | PRKCA |
| rs16966598 | rs1985152 | AA_int_oo | SNP1xSNP2  (C, G) | C<G (0.15) | G<A (0.37) | 3.8X10^-7^ | 0.84 (0.79- 0.9) | 1.9X10^-7^ | 0.84 (0.78-0.9) | NOS2 | JAZF1 |
| rs1523303 | rs2266967 | AA_int_ro | SNP1xSNP2  (G, A) | A<G (0.38) | A<C (0.5) | 6.6X10^-7^ | 1.07 (1.04- 1.1) | 3.6X10^-7^ | 1.08 (1.05-1.11) | TGFA | MAPK1 |
| rs1649949 | rs16958619 | DD_int_oo | (AG/AA+ AC/AA) vs. others | A<G (0.19) | A<C (0.13) | 8.7X10^-7^ | 1.35 (1.2- 1.51) | 6.8X10^-7^ | 1.36 (1.20-1.53) | NRG3 | CDH13 |
| rs1250240 | rs174776 | DR_int_ro | (GG+ AA) vs. others | A<G (0.26) | A<G (0.11) | 2.4X10^-9^ | 2.96 (2.07- 4.24) | 1.1X10^-6^ | 2.59 (1.76-3.79) | FN1 | KLK3 |
| rs4512403 | rs9517971 | DR_int_oo | (GA/GG+ CC) vs. others | G<A (0.17) | C<A (0.48) | 1.6X10^-6^ | 0.71 (0.61- 0.81) | 1.8X10^-6^ | 0.7 (0.61-0.81) | EIF3H:RP11-536K17.1 | PCCA |
| rs12143015 | rs1518887 | AA_int_oo | SNP1xSNP2  (A, G) | A<G (0.22) | G<A (0.39) | 7.9X10^-7^ | 1.13 (1.08- 1.18) | 4.0X10^-6^ | 1.12 (1.07-1.18) | RASAL2 | LINC01811 |
| rs4148853 | rs905557 | AA_int_oo | SNP1xSNP2  (A, A) | A<G (0.19) | A<G (0.13) | 9.3X10^-7^ | 1.26 (1.15- 1.39) | 4.3X10^-6^ | 1.25 (1.14-1.38) | ABCB8 | OXR1 |
| rs1331981 | rs7326449 | DD_int_rr | (GG+ AA) vs. others | A<G (0.07) | G<A (0.21) | 3.8X10^-7^ | 0.84 (0.78- 0.9) | 5.7X10^-6^ | 0.85 (0.79-0.91) | LINC02544 | COL4A2 |
| rs1483192 | rs174776 | AA_int_ro | SNP1xSNP2  (G, A) | A<G (0.49) | A<G (0.11) | 3.2X10^-7^ | 1.14 (1.08- 1.19) | 6.0X10^-6^ | 0.87 (0.82-0.93) | TMSB4X | KLK3 |
| rs11214133 | rs9521760 | AA_int_oo | SNP1xSNP2  (A, A) | A<C (0.12) | A<G (0.29) | 1.8X10^-6^ | 1.2 (1.11- 1.29) | 8.9X10^-5^ | 1.17 (1.08-1.26) | BCO2 | COL4A2 |
| rs12126922 | rs1654513 | AA_int_or | SNP1xSNP2  (G, A) | G<C (0.39) | G<A (0.34) | 7.8X10^-6^ | 1.07 (1.04- 1.1) | 1.1X10^-4^ | 1.06 (1.03-1.09) | KIF26B | KLKP1 |
| rs1433165 | rs7446 | DD_int_oo | (AG/AA+ AG/AA) vs. others | A<G (0.41) | A<G (0.31) | 4.9X10^-8^ | 1.22 (1.13- 1.31) | 1.4X10^-4^ | 1.16 (1.08-1.25) | ANGPT1 | KPNA3 |
| rs12619116 | rs994029 | DD_int_oo | (GA/GG+ GA/GG) vs. others | G<A (0.14) | G<A (0.38) | 5.9X10^-6^ | 0.8 (0.72- 0.88) | 2.1X10^-4^ | 0.83 (0.75-0.91) | COL4A3 | GAS1:RP11-395D3.1 |
| rs7593402 | rs994029 | DD_int_or | (GA/GG+ AA)  vs. others | G<A (0.13) | G<A (0.38) | 8.8X10^-6^ | 1.29 (1.15- 1.45) | 2.6X10^-4^ | 1.24 (1.11-1.40) | ARHGAP25 | GAS1:RP11-395D3.1 |
| rs7446 | rs4919687 | AA_int_oo | SNP1xSNP2  (A, A) | A<G (0.31) | A<G (0.29) | 5.2X10^-7^ | 1.13 (1.08- 1.18) | 5.1X10^-4^ | 1.10 (1.04-1.15) | KPNA3 | CYP17A1 |
| rs10827164 | rs2569735 | DD_int_or | (GA/GG+ GG)  vs. others | G<A (0.36) | A<G (0.12) | 1.7X10^-9^ | 0.81 (0.76- 0.87) | 8.4X10^-4^ | 0.88 (0.82-0.95) | ITGB1 | KLK3 |
| rs219154 | rs17632542 | DD_int_oo | (AG/AA+ GA/GG)  vs. others | A<G (0.31) | G<A (0.06) | 3.1x10^-15^ | 1.7 (1.49- 1.94) | 1.4X10^-3^ | 1.34 (1.12-1.60) | LTBP1 | KLK3 |
| rs174776 | rs2361634 | AA_int_rr | SNP1xSNP2  (G, A) | A<G (0.11) | G<A (0.07) | 4.3X10^-9^ | 0.92 (0.90- 0.95) | 2.0X10^-3^ | 0.95 (0.92-0.98) | KLK3 | AR |
| rs4878112 | - | AA_M1_int_r1 | SNP1  (G) | A<G (0.25) | - | 2.5x10^-4^ | 0.90 (0.85-0.95) | 3.0X10^-3^ | 0.92 (0.87-0.97) | DAPK1 | - |
| rs4878112 | rs17632542 | AA_M1_int_r1 | SNP1xSNP2  (G, A) | A<G (0.25) | A<G (0.46) | 1.9x10^-14^ | 1.27 (1.19- 1.34) | 4.8x10^-2^ | 1.09 (1.00-1.19) | DAPK1 | KLK3 |
| rs1060263 | rs4802755 | DR_int_oo | (GA/GG+ GG)  vs. others | G<A (0.17) | G<A (0.35) | 1.7X10^-7^ | 1.40 (1.24- 1.59) | 3.0X10^-3^ | 1.22 (1.07-1.40) | TOX | KLK3 |
| rs1010 | rs2271095 | AA_int_rr | SNP1xSNP2  (A, A) | G<A (0.4) | G<A (0.35) | 1.6X10^-8^ | 1.08 (1.05- 1.11) | 4.0X10^-3^ | 1.04 (1.01-1.07) | VAMP8 | KLK3 |
| rs1463592 | rs2271095 | AA_int_oo | SNP1xSNP2  (A, A) | A<C (0.22) | A<G (0.07) | 2.3X10^-6^ | 0.88 (0.83- 0.93) | 1.1X10^-2^ | 0.93 (0.87-0.98) | SARNP:ORMDL2:DNAJC14:LOC440104:MMP19:WIB | KLK3 |
| rs390993 | rs473640 | AA_Full | SNP1 | A<G  (0.25) | G<A  (0.18) | 1.9X10^-2^ | 0.92 (0.86-0.99) | 2.0X10^-2^ | 0.92 (0.86-0.99) | RIPK2 | NOS1 |
|  |  |  | SNP2 |  |  | 1.1X10^-3^ | 0.87 (0.8-0.95) | 1.6X10^-3^ | 0.87 (0.80-0.95) |  |  |
|  |  |  | SNP1xSNP2 |  |  | 2.8X10^-7^ | 1.31 (1.18-1.45) | 1.5X10^-6^ | 1.29 (1.16-1.44) |  |  |

^a^ SIPI pattern interpretation: The first 2 letters represent inheritance modes of the first SNP (or SNP1) and the 2^nd^ SNPs (or SNP2); mode (A, additive mode; D, dominant mode; R, recessive mode; “(SNP1 mode)(SNP2 mode)_int_(SNP1 code)(SNP2 code)” is for interaction-only pattern; “Mϕ_int_”, interaction plus one main effect of SNPϕ; SNP code (o, original coding based on the minor allele; r, reverse coding; r1: reverse coding for SNP1 (SNP2 with original coding)

^b^Uni-pair: model with only 1 SNP pair; Multi-pair: model with 24 SNP pair; all models adjusted for study site and first six principal components

Abbreviations: CI, confidence interval; Dis, discovery set; MAF, minor allele frequency; Maj, major allele; Min, minor allele; OR, odds ratio; Val, validation set.

Table S4. Performance of multi-pair models (24 pairs and 12 pairs) associated with prostate cancer aggressiveness

1. 24-pair model

| Risk score^a^ | Sample size (%) | PCa aggr % (95% CI)^b^ | OR (95% CI)^c^ | p-value |
| --- | --- | --- | --- | --- |
| 0-0.9 | 200 ( 1.0) | 7.0 ( 3.5-10.5) | 0.27 (0.16-0.47) | 4.1x10^-6^ |
| 1-9.9 | 1808 ( 9.0) | 13.7 (12.1-15.3) | 0.55 (0.48-0.64) | 6.3 x10^-16^ |
| 10-24.9 | 3014 (15.0) | 16.7 (15.4-18.0) | 0.70 (0.62-0.78) | 6.8 x10^-11^ |
| 25-74.9 | 10045 (50.0) | 21.9 (21.1-22.7) | 1 | - |
| 75-89.9 | 3013 (15.0) | 27.2 (25.6-28.8) | 1.37 (1.24-1.51) | 1.8 x10^-10^ |
| 90-98.9 | 1809 ( 9.0) | 36.2 (34.0-38.4) | 1.97 (1.77-2.20) | 4.0 x10^-33^ |
| 99-100 | 199 ( 1.0) | 49.8 (42.8-56.7) | 3.65 (2.71-4.91) | 1.2 x10^-17^ |

1. 12-pair model

| Risk score^a^ | Sample size (%) | PCa aggr % (95% CI)^b^ | OR (95% CI)^c^ | p-value |
| --- | --- | --- | --- | --- |
| 0-0.9 | 201 ( 1.0) | 11.9 (7.5-16.4) | 0.51 (0.33-0.78) | 2.3 x10^-3^ |
| 1-9.9 | 1803 ( 9.0) | 15.0 (13.3-16.6) | 0.60 (0.52-0.69) | 1.3 x10^-12^ |
| 10-24.9 | 3008 (15.0) | 18.1 (16.7-19.4) | 0.77 (0.69-0.86) | 1.1 x10^-6^ |
| 25-74.9 | 10039 (50.0) | 21.8 (21.0-22.6) | 1 | - |
| 75-89.9 | 3007 (15.0) | 27.6 (26.0-29.2) | 1.38 (1.25-1.52) | 1.7 x10^-10^ |
| 90-98.9 | 1805 ( 9.0) | 32.7 (30.5-34.9) | 1.73 (1.54-1.93) | 2.9 x10^-21^ |
| 99-100 | 201 ( 1.0) | 45.3 (38.4-52.2) | 3.06 (2.28-4.11) | 2.0 x10^-13^ |

^a^ 23-pair model selected based on stepwise p<0.01. 12-pair model selected based on stepwise p<1x10^-5^. Risk score’s cut-points were based on the percentiles (<1, 1-10, 10-5, 25-75, 75-90, 90-99 and ≥99%) of the predicted probabilities in the combined set, N=20064.

^b^ Prevalence of prostate cancer aggressiveness, 95% exact confidence interval

^c^ OR: odds ratio of prostate cancer aggressiveness; CI: confidence interval; model adjusted for study sties and six principal components

Table S5. Model performance comparisons

| Model^a^ | Model contents | AUC^b^ | R^2^ | BIC^c^ | p_AUC^b^  (vs. 24-pair) | p_AUC^b^  (vs. 12-pair) |
| --- | --- | --- | --- | --- | --- | --- |
| Individual-1 | 1 verified SNP | 0.668 | 0.095 | 20364.6 | 6.5x10^-27^ | 1.7x10^-17^ |
| Individual-11 | 11 hub SNPs in clusters | 0.672 | 0.097 | 20423.2 | 2.6x10^-24^ | 2.0x10^-12^ |
| Individual-24 | 24 SNPs in the 12-pair model | 0.680 | 0.107 | 20424.2 | - | 2.2x10^-5^ |
| Individual-42 | 42 SNPs in the 24-pair model | 0.686 | 0.113 | 20505.3 | 1.1x10^-7^ | - |
| 12-pair | 12 SNP-interaction pairs | 0.687 | 0.115 | 20187.6 | 2.7x10^-8^ | **-** |
| 24-pair | 24 SNP-interaction pairs | **0.696** | **0.126** | **20171.1** | **-** | **-** |

^a^Model performance was compared based the same set of participants with complete SNP information in all three models, n=20,062

^b^AUC (area under the receiver operating characteristic curve), p_AUC: compare AUC values between the individual effect model with the multi-pair interaction model

^c^BIC: Bayesian information criterion. Bootstrap confidence intervals of AUC for the 12- and 23-pair models were 0.679-0.695 and 0.684-0.701 based on 1000 runs.

Table S6. List of the 24 significant expression quantitative trait loci (eQTL) results

| SNP-int pair^a^ | eQTL  Model^b^ | eQTL  p-value | SNP-int  p-value | Gene_Exp | SNP-int gene pair  (gene1_gene2) | PCa-aggr SNP pair^c^ |
| --- | --- | --- | --- | --- | --- | --- |
| rs2296326_rs17632542 | AA_int_or | 2.5 x10^-14^ | 1.1x10^-11^ | *CHURC1* | *RAB15:CHURC1-FNTB_KLK3* | No |
| rs7634934_rs17632542 | AA_int_or | 5.2x10^-10^ | 3.3x10^-14^ | *EFHB* | *RAB5A_KLK3* | Yes |
| rs10154906_rs17632542 | AA_int_or | 1.4x10^-9^ | 1.2x10^-14^ | *EPHB1* | *EPHB1_KLK3* | Yes |
| rs7221407_rs17632542 | AA_int_oo | 3.0x10^-9^ | 1.8x10^-13^ | *JAK1* | *RAB11FIP4_KLK3* | No |
| rs2163417_rs17632542 | AA_int_oo | 3.1x10^-9^ | 3.2x10^-13^ | *JAK1* | *TGFBR2_KLK3* | No |
| rs11466561_rs17632542 | AA_int_oo | 6.2x10^-9^ | 3.4x10^-13^ | *NEBL* | *TGFBR3_KLK3* | No |
| rs17131560_rs17632542 | AA_int_oo | 6.4x10^-9^ | 5.0x10^-13^ | *NEBL* | *TGFBR3_KLK3* | No |
| rs709937_rs17632542 | AA_int_oo | 7.6x10^-9^ | 1.9x10^-11^ | *MGST1* | *NGEF_KLK3* | No |
| rs34358951_rs2569735 | AA_int_oo | 7.1 x10^-13^ | 1.9x10^-8^ | *USP40* | *COL4A3_KLK3* | No |
| rs72656724_rs2569735 | AA_int_oo | 1.2 x10^-10^ | 4.0x10^-8^ | *HNF1B* | *CDKN2B-AS1_KLK3* | No |
| rs4582031_rs2569735 | AA_int_oo | 3.2 x10^-10^ | 9.6x10^-9^ | *FGF1* | *EPHB3_KLK3* | Yes |
| rs2912791_rs1058205 | AA_M1_int_r1 | 3.4 x10^-10^ | 2.1x10^-7^ | *KLK3* | *FGFR2_KLK3* | Yes |
| rs970842_rs1058205 | AA_int_oo | 9.5 x10^-10^ | 3.5x10^-7^ | *ITGB6* | *LINC01811_KLK3* | Yes |
| rs7634934_rs174776 | AA_int_or | 8.2 x10^-10^ | 1.9x10^-6^ | *EFHB* | *RAB5A_KLK3* | Yes |
| rs7224135_rs174776 | AA_int_ro | 2.5x10^-9^ | 9.5x10^-7^ | *SRD5A2* | *CAVIN1_KLK3* | Yes |
| rs2938828_rs174776 | AA_int_ro | 9.8x10^-9^ | 4.1x10^-6^ | *GMPR* | *LINC02199_KLK3* | Yes |
| rs7626555_rs17632542 | AA_int_rr | 1.1x10^-9^ | 4.6x10^-15^ | *PDCD6IP* | *PDCD6IP_KLK3* | Yes |
| rs7626555_rs1058205 | AA_int_or | 2.2x10^-9^ | 3.4x10^-7^ | *PDCD6IP* | *PDCD6IP_KLK3* | Yes |
| rs7626555_rs2569735 | AA_int_or | 2.4x10^-9^ | 1.4x10^-8^ | *PDCD6IP* | *PDCD6IP_KLK3* | Yes |
| rs7870707_rs1058205 | AA_int_oo | 5.8 x10^-12^ | 1.3x10^-7^ | *TMEM185B* | *MED22_KLK3* | Yes |
| rs7870707_rs1058205 | AA_int_oo | 1.9x10^-9^ | 1.3x10^-7^ | *RALB* | *MED22_KLK3* | Yes |
| rs7870707_rs1058205 | AA_int_oo | 6.0x10^-9^ | 1.3x10^-7^ | *CLASP1* | *MED22_KLK3* | Yes |
| rs995633_rs1058205 | AA_int_oo | 8.2 x10^-10^ | 1.1x10^-7^ | *SLC25A21* | *FHIT_KLK3* | Yes |
| rs995633_rs2569735 | AA_int_oo | 1.4x10^-9^ | 1.9x10^-9^ | *SLC25A21* | *FHIT_KLK3* | Yes |

^a^ eQTL tests had P-value <1.1x10^-8^, Bonferroni criteria

^b^Pattern interpretation: The first 2 letters represent inheritance modes of the first SNP (or SNP1) and the 2^nd^ SNPs (or SNP2); A, additive mode; “(SNP1 mode)(SNP2 mode)_int_(SNP1 code)(SNP2 code)” is for interaction-only pattern; “Mϕ_int_”, interaction plus one main effect of SNPϕ; SNP code (o, original coding based on the minor allele; r, reverse coding; r1: reverse coding for SNP1 (SNP2 with original coding)

^c^In the list of 3144 SNP pairs, which were significantly associated with prostate cancer aggressiveness

Abbreviations: SNP-int pair, SNP-interaction pair, Gene_Exp, gene expression, SNP-int gene pair: genes for the SNP-SNP interaction pair

Table S7. Pathway information for the top 3144 SNP-SNP interaction pairs

| Pathway-1 | Pathway-2 | Number of SNP pairs (%) |
| --- | --- | --- |
| Androgen | Angiogenesis | 1543 (49.1) |
| Androgen | Mitochondria | 1138 (36.2) |
| Androgen | miRNA | 228 ( 7.3) |
| Androgen | Androgen | 200 ( 6.4) |
| miRNA | Angiogenesis | 12 ( 0.4) |
| miRNA | Mitochondria | 8 ( 0.3) |
| Angiogenesis | Angiogenesis | 6 ( 0.2) |
| Mitochondria | Angiogenesis | 6 ( 0.2) |
| Mitochondria | Mitochondria | 3 ( 0.1) |
| Total |  | 3144 (100) |

Table S8. Brief literature review of *KLK3* SNPs and prostate cancer

| **SNP**^a^ | **P value** | **Phenotype**^b^ | **Population** | **Citation** |
| --- | --- | --- | --- | --- |
| rs2735839 | 1.5x10^-18^ | Prostate cancer | White (n= 1,854) | Eeles et al.^5^ |
| rs17632542 | 9.0x10^−40^ | PSA | Whites (Iceland [n= 6,078] & UK [n=435]) | Gudmundsson et al.^6^ |
|  | 2.7x10^−9^ |  |  |  |
| rs2735839 | 6.5x10^-37^ | PSA | Chinese (n=3,495) | Sun et al.^7^ |
| rs17632542 | 1.0x10^-7^ | Prostate cancer (early onset) | White (n=931) | Lange et al.^8^ |
| rs17632542 | 2.3x10^-28^ | Prostate cancer | White (n=6,241) | Knipe et al.^9^ |
| rs1058205 | 8.3x10^-21^ | PSA | Japanese (n= 2,388) | Terao et al.^10^ |
| rs2735839 | 5.7x10^-9^ | Prostate cancer | Non-Hispanic white, Latino, East Asian, & African American (n=46,378) | Hoffmann et al.^11^ |
| rs2659124 | 1.9x10^-12^ |  |  |  |
| rs17632542 | 2.4x10^-340^ | PSA | Non-Hispanic white, Latino, East Asian,  & African American (n=47,118) | Hoffmann et al.^12^ |
| rs2739472 | 1.5x10^-77^ |  |  |  |
| rs6070 | 3.9x10^-11^ |  |  |  |
| rs266849 | 9.2x10^-165^ |  |  |  |
| rs266868 | 5.0x10^-1^ |  |  |  |
| rs2735839 | 7.6x10^−20^ | PSA | Korean (n=4,124) | Choe et al.^13^ |
| rs266849 | 1.8x10^−14^ |  |  |  |
| rs2411332 | 2.4x10^−9^ |  |  |  |
| rs2735839 | 3.3x10^-47^ | Prostate cancer | White (46,939 cases and  27,910 controls) | Schumacher et al.^14^ |
| rs2659124 | 1.7x10^-8^ | Prostate cancer | Japanese (9,906 cases and  83,943 controls) | Takata et al.^15^ |
| rs2735839 | 2.9x10^-9^ | Prostate cancer |  |  |
| rs1058205 | 4.0x10^-4^ | PSA | Blacks (n=1,130) | Bensen, et al.^16^ |
| rs1058205 | 2.0x10^-2^ | Gleason score | Whites (n=1,128) | Bensen, et al.^16^ |
| rs266870 | 1.0x10^-3^ | PSA | Blacks (n=1,130) | Bensen, et al.^16^ |
| rs266870 | 1.0x10^-2^ | Stage | Whites (n=1,128) | Bensen, et al.^16^ |
| rs266870 | 9.0x10^-3^ | Gleason score | Whites (n=1,128) | Bensen, et al.^16^ |
| rs2735839 | 2.0x10^-3^ | PSA | Blacks (n=1,130) | Bensen, et al.^16^ |
| rs2735839 | 2.0x10^-2^ | Gleason score | Whites (n=1,128) | Bensen, et al.^16^ |
| rs62113212 | 5.9x10^-9^ | Gleason score | Whites (N=12,508) | Berndt, et al.^17^ |
| rs62113212 | 6.0x10^-9^ | Prostate cancer aggressiveness | Whites (N=12,508) | Berndt et al.^17^ |

^a^rs1058205 and rs2735839 were in high linkage disequilibrium (r^2^≥0.8); rs17632542 and rs62113212 were in complete linkage disequilibrium (r^2^=1)

^b^PSA: Prostate-specific antigen levels

Table S9.SNP-SNP interaction patterns/models of the AA9int and SIPI approaches (complicated models with ≥1 main effects)

| Model structure ^b^ | Add_Add (AA)^a^ | Dom_Dom (DD)^a^ | Dom_Rec (DR)^a^ | Rec_Dom (RD)^a^ | Rec-Rec (RR) ^a^ |
| --- | --- | --- | --- | --- | --- |
| Full | 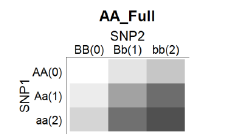 | 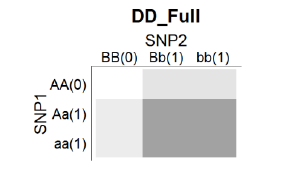 | 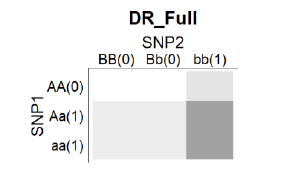 | 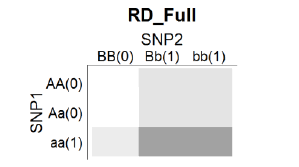 | 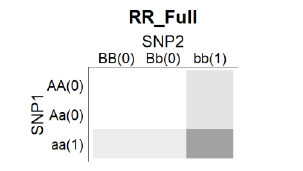 |
|  | **aSNP1_o_ + aSNP2_o_ + aSNP1_o_ x aSNP2_o_** | **dSNP1_o_ +dSNP2_o_ + dSNP1_o_ x dSNP2_o_** | **dSNP1_o_ + rSNP2_o_ + dSNP1_o_ x rSNP2_o_** | **rSNP1_o_ + dSNP2_o_ + rSNP1_o_ x dSNP2_o_** | **rSNP1_o_ + rSNP2_o_ + rSNP1_o_ x rSNP2_o_** |
| M1_int_o_1_ | 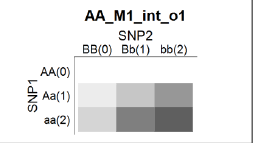 | 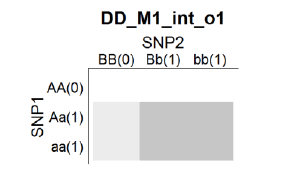 | 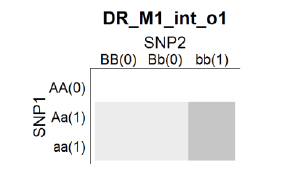 | 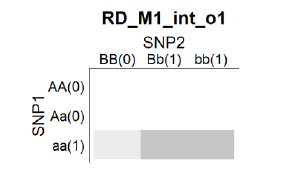 | 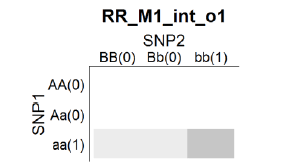 |
|  | **aSNP1_o_ + aSNP1_o_ x aSNP2_o_** | **dSNP1_o_ + dSNP1_o_ x dSNP2_o_** | **dSNP1_o_ + dSNP1_o_ x rSNP2_o_** | **rSNP1_o_ + rSNP1_o_ x dSNP2_o_** | **rSNP1_o_ + rSNP1_o_ x rSNP2_o_** |
| M1_int_r_1_ | 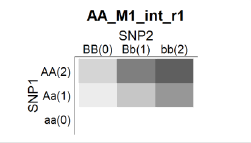 | 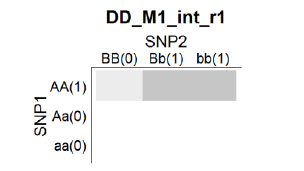 | 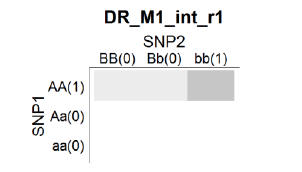 | 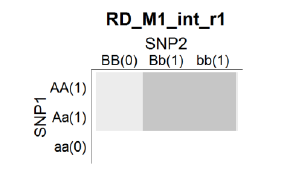 | 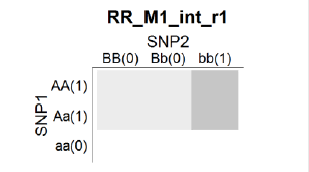 |
|  | **aSNP1_r_ + aSNP1_r_ x aSNP2_o_** | **dSNP1_r_ + dSNP1_r_ x dSNP2_o_** | **dSNP1_r_ + dSNP1_r_ x rSNP2_o_** | **rSNP1_r_ + rSNP1_r_ x dSNP2_o_** | **rSNP1_r_ + rSNP1_r_ x rSNP2_o_** |
| M2_int_o_2_ | 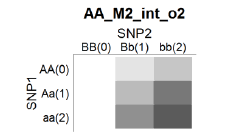 | 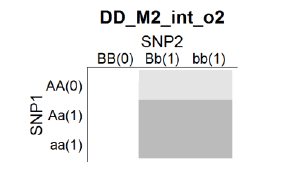 | 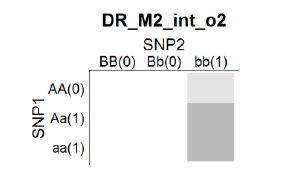 | 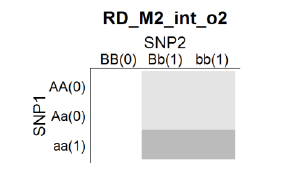 | 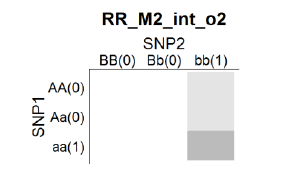 |
|  | **aSNP2_o_ +aSNP1_o_ x aSNP2_o_** | **dSNP2_o_ + dSNP1_o_ x dSNP2_o_** | **rSNP2_o_ + dSNP1_o_ x rSNP2_o_** | **dSNP2_o_ + rSNP1_o_ x dSNP2_o_** | **rSNP2_o_ + rSNP1_o_ x rSNP2_o_** |
| M2_int_r_2_ | 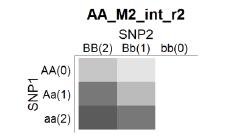 | 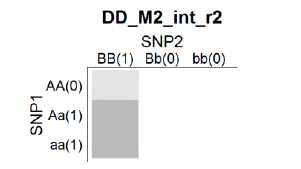 | 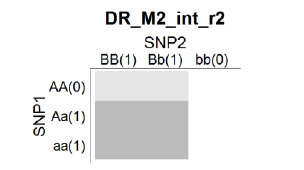 | 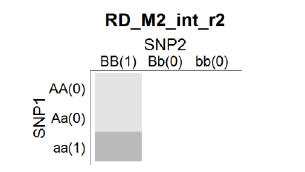 | 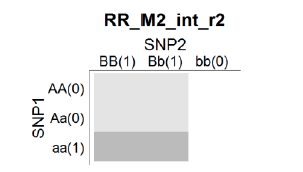 |
|  | **aSNP2_r_ +aSNP1_o_ x aSNP2_r_** | **dSNP2_r_ + dSNP1_o_ x dSNP2_r_** | **rSNP2_r_ + dSNP1_o_ x rSNP2_r_** | **dSNP2_r_ + rSNP1_o_ x dSNP2_r_** | **rSNP2_r_ + rSNP1_o_ x rSNP2_r_** |

^a^ AA9int (Additive-additive 9 interaction-model approach) tested for 9 additive-additive models; SIPI (SNP Interaction Pattern Identifier), Dom: dominant, Rec: recessive, Add: additive The labels of two axes are ‘genotype (coding).’ A lowercase letter denotes the minor allele, and an uppercase letter denotes the major allele. Color levels inside the cells represent present outcome proportions. The darker the color, the higher the outcome proportion. These are example patterns based on both minor alleles as risk alleles.

^b^ Full: full interaction model with two main effects plus an interaction; M1+int: main effect of SNP1 plus an interaction; M2+int: main effect of SNP2 plus an interaction; and (4) Int-only: an interaction only. The formula includes covariates in the model. A letter in front of SNP1/SNP2 indicates an inheritance mode, and a subscript indicates a coding type.

_o_1_, _r_1_: count of minor allele (original coding), and count of major allele (reverse coding) of SNP_1_; _o_2_, _r_2_: count of minor allele (original coding), and count of major allele (reverse coding) of SNP_2_;_oo, _or, _ro, _rr: based on original-original, original-reverse, reverse-original and reverse-reverse coding for SNP1 and SNP2.

Table S10. SNP-SNP interaction patterns/models of the AA9int and SIPI approaches (Interaction-only models)

| Model structure ^b^ | Add_Add (AA)^a^ | Dom_Dom (DD)^a^ | Dom_Rec (DR)^a^ | Rec_Dom (RD)^a^ | Rec-Rec (RR) ^a^ |
| --- | --- | --- | --- | --- | --- |
| int_oo | 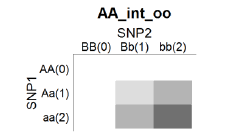 | 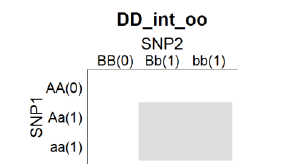 | 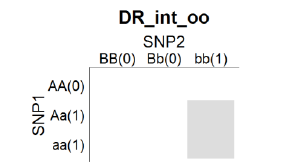 | 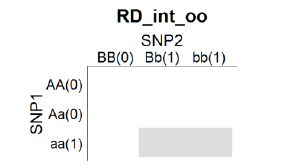 | 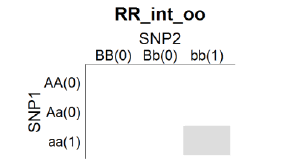 |
|  | **aSNP1_o_ x aSNP2_o_** | **dSNP1_o_ x dSNP2_o_** | **dSNP1_o_ x rSNP2_o_** | **rSNP1_o_ x dSNP2_o_** | **rSNP1_o_ x rSNP2_o_** |
| int_or | 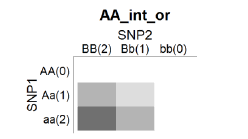 | 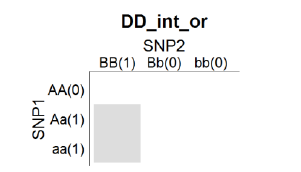 | 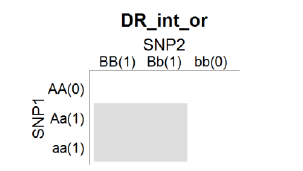 | 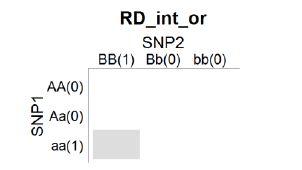 | 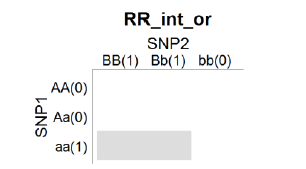 |
|  | **aSNP1_o_ x aSNP2_r_** | **dSNP1_o_ x dSNP2_r_** | **dSNP1_o_ x rSNP2_r_** | **rSNP1_o_ x dSNP2_r_** | **rSNP1_o_ x rSNP2_r_** |
| int_ro | 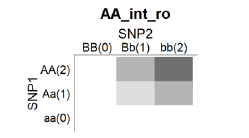 | 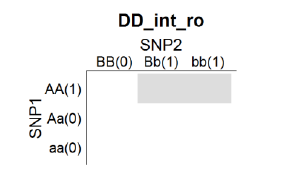 | 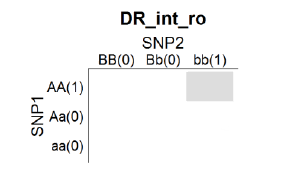 | 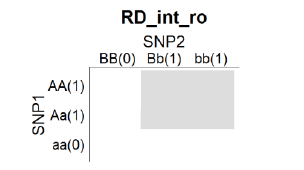 | 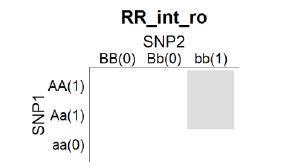 |
|  | **aSNP1_r_ x aSNP2_o_** | **dSNP1_r_ x dSNP2_o_** | **dSNP1_r_ x rSNP2_o_** | **rSNP1_r_ x dSNP2_o_** | **rSNP1_r_ x rSNP2_o_** |
| int_rr | **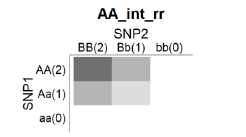** | 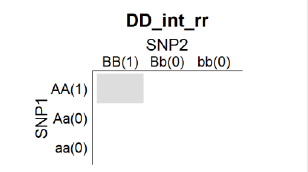 | 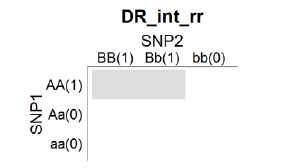 | 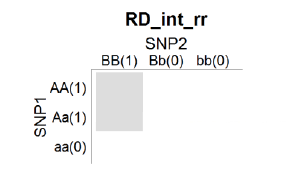 | 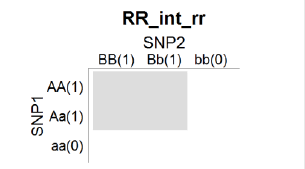 |
|  | **aSNP1_r_ x aSNP2_r_** | **dSNP1_r_ x dSNP2_r_** | **dSNP1_r_ x rSNP2_r_** | **rSNP1_r_ x dSNP2_r_** | **rSNP1_r_ x rSNP2_r_** |

^a^ AA9int (Additive-additive 9 interaction-model approach) tested for 9 additive-additive models; SIPI (SNP Interaction Pattern Identifier), Dom: dominant, Rec: recessive, Add: additive The labels of two axes are ‘genotype (coding).’ A lowercase letter denotes the minor allele, and an uppercase letter denotes the major allele. Color levels inside the cells represent present outcome proportions. The darker the color, the higher the outcome proportion. These are example patterns based on both minor alleles as risk alleles.

^b^ Full: full interaction model with two main effects plus an interaction; M1+int: main effect of SNP1 plus an interaction; M2+int: main effect of SNP2 plus an interaction; and (4) Int-only: an interaction only. The formula includes covariates in the model. A letter in front of SNP1/SNP2 indicates an inheritance mode, and a subscript indicates a coding type.

_o_1_, _r_1_: count of minor allele (original coding), and count of major allele (reverse coding) of SNP_1_; _o_2_, _r_2_: count of minor allele (original coding), and count of major allele (reverse coding) of SNP_2_;_oo, _or, _ro, _rr: based on original-original, original-reverse, reverse-original and reverse-reverse coding for SNP1 and SNP2.

Figure S1. Linkage disequilibrium plot for the *KLK3* SNPs


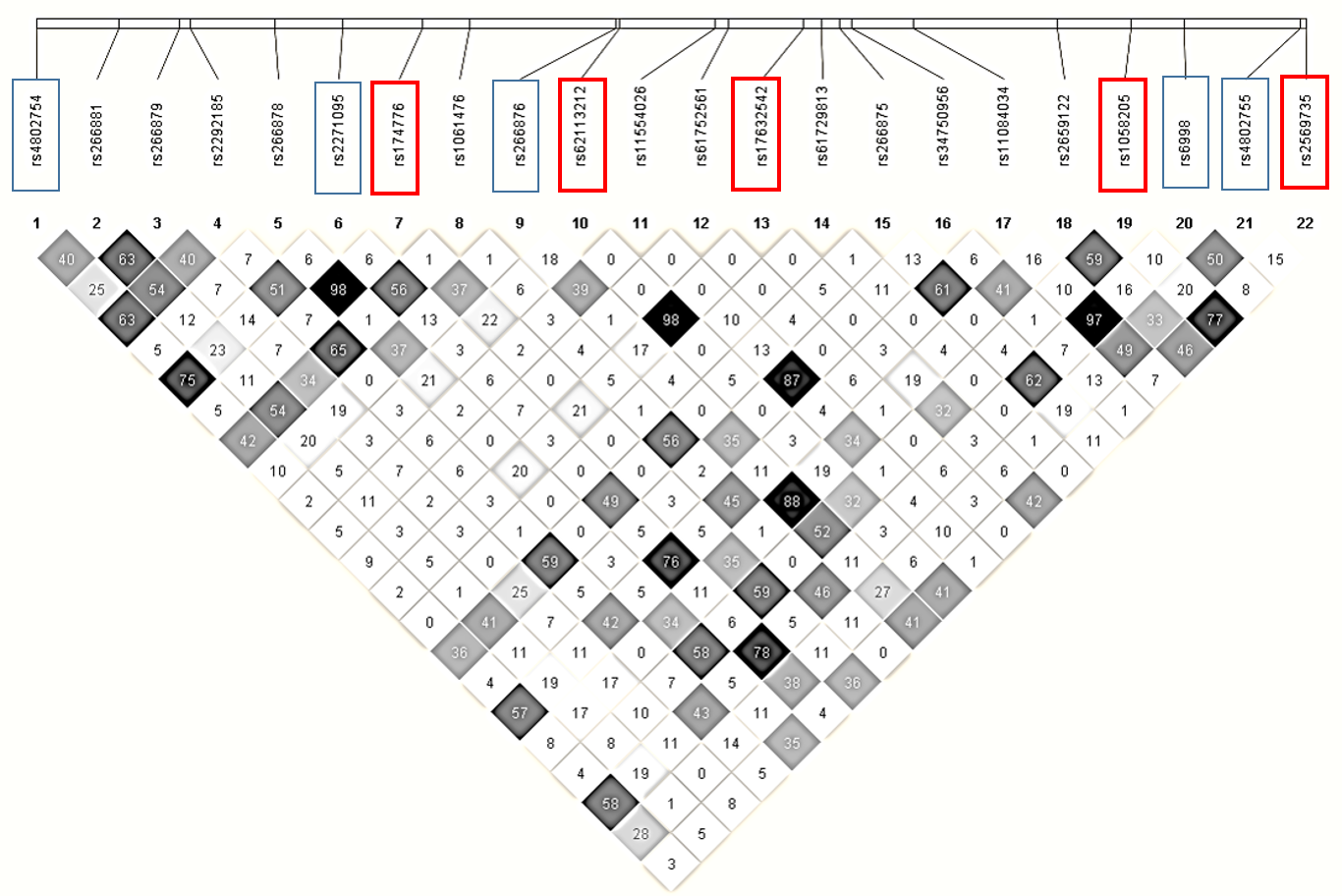


Note: The values in the plot are r^2^. The darker the color, the stronger the linkage disequilibrium. The top 4 SNPs in the SNP-SNP interactions are marked with a rectangle.

Figure S2. SNP-SNP interactions of rs390993 and rs473640 associated with prostate cancer aggressiveness


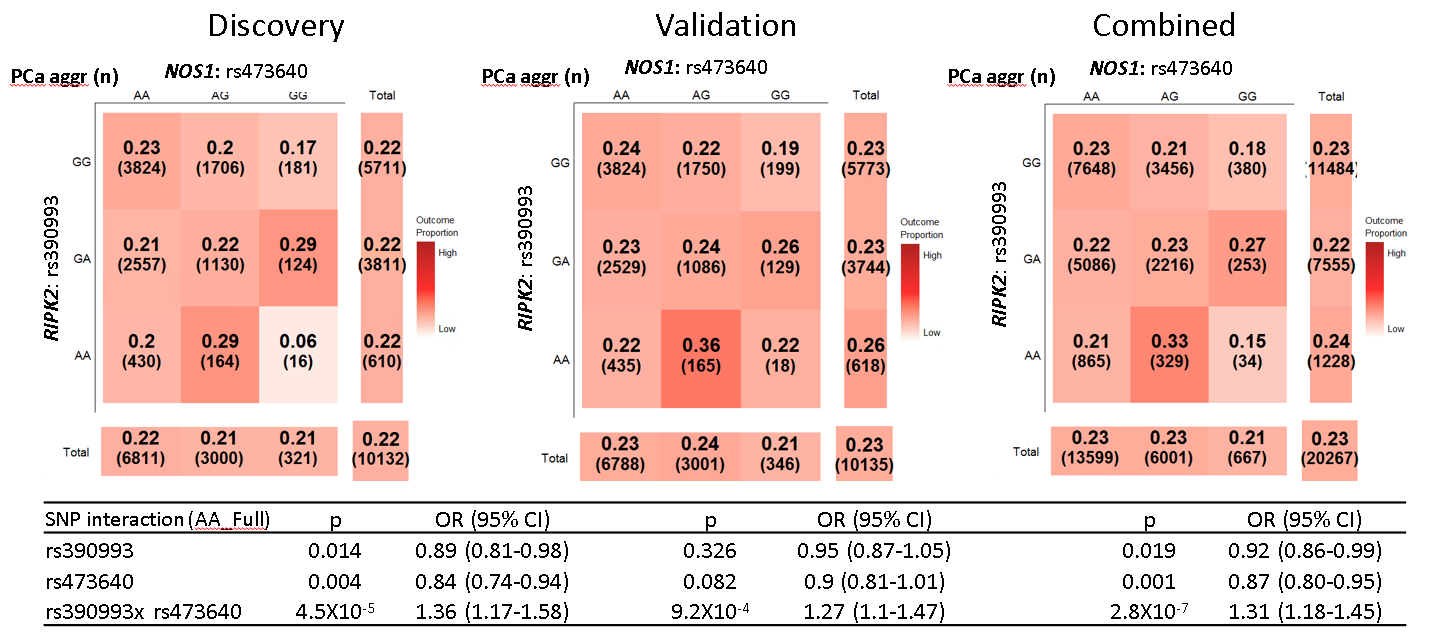


Note: PCa aggr (n): prostate cancer aggressiveness % (sample size in the genotype combination); OR: odds ratio of prostate cancer aggerssiveness, CI: confidence interval

Figure S3. Significance levels of the SNP-SNP interaction pairs in the combined set for the top 66,619 SNP pairs with a p<1x10^-3^ in the discovery set


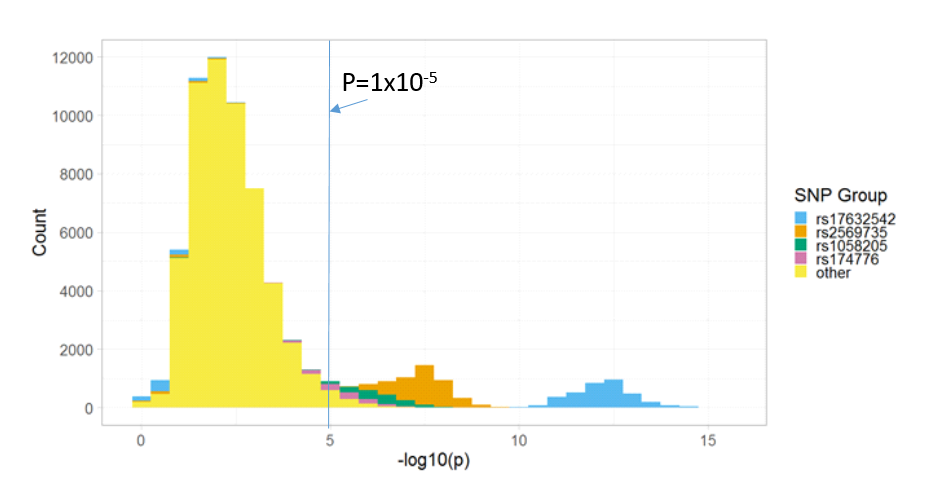


Figure S4. Example of SNP-SNP interactions associated with prostate cancer aggressiveness with a complicated interaction pattern (one main effect and an interaction term)


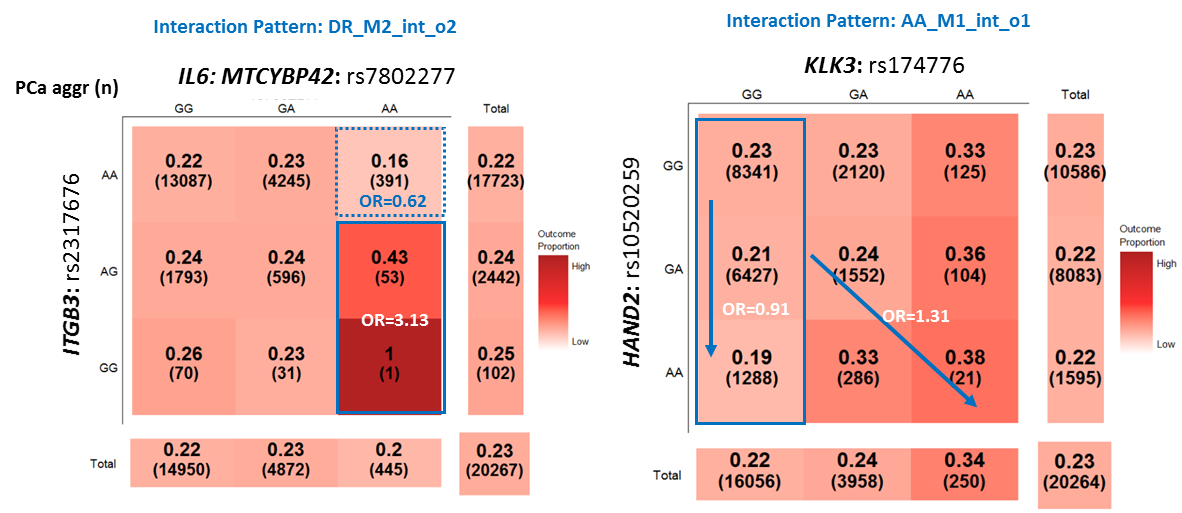


Note: PCa aggr (n): prostate cancer aggressiveness % (sample size in the genotype combination);

“(SNP1 mode)(SNP2 mode)**_** Mϕ_int**_**(SNPϕ code)”: interaction plus one main effect of SNPϕ; modes (A: additive, D: dominant, R: recessive); code (o: original coding based on the minor allele, r: reverse); _o_1_, _r_1_: original code SNP1 and reverse coding of SNP1 (SNP2 had an original coding); o_2_, _r_2_: original code, and reverse coding of SNP2 (SNP1 had original coding); OR: odds ratio of PCa aggressiveness adjusted for study sties and six principal components for population stratification

Figure S5. Plot of -log10 p-value of the expression quantitative trait loci (eQTL) tests using The Cancer Genome Atlas (TCGA) prostate cancer data (n=231)


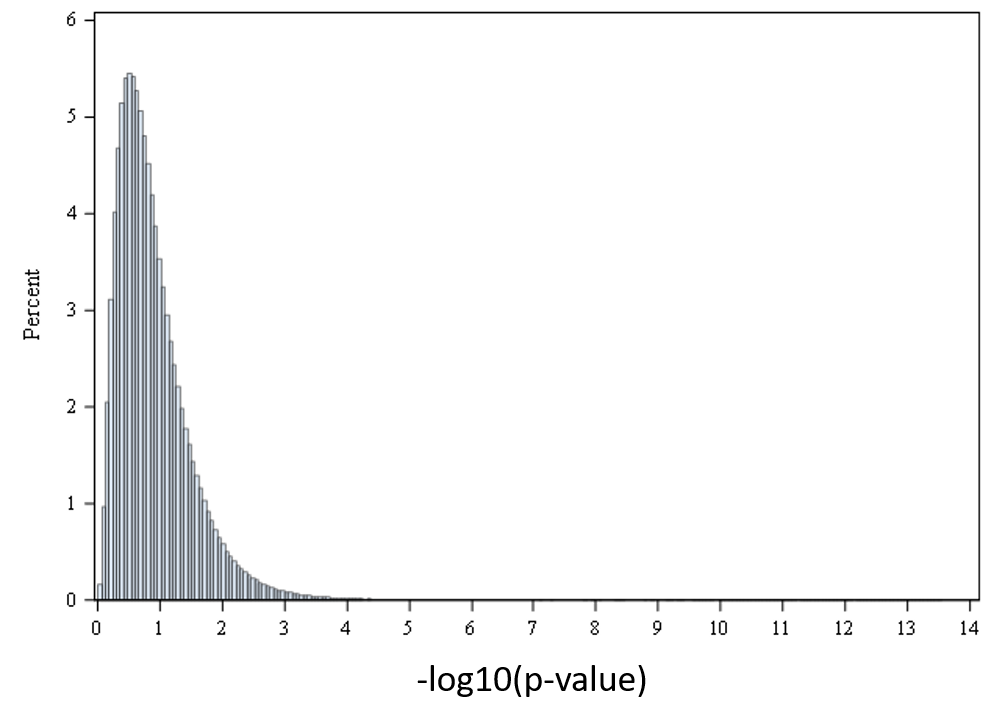


Note: Based on the selected 3,056 SNP pairs and expression levels of 1,459 genes of the four candidate pathways

Figure S6. *SRD5A2* expression by the genotype combinations of rs7224135 (*CAVIN1*) and rs174776 (*KLK3*)

PRACTICAL Results

TCGA Results


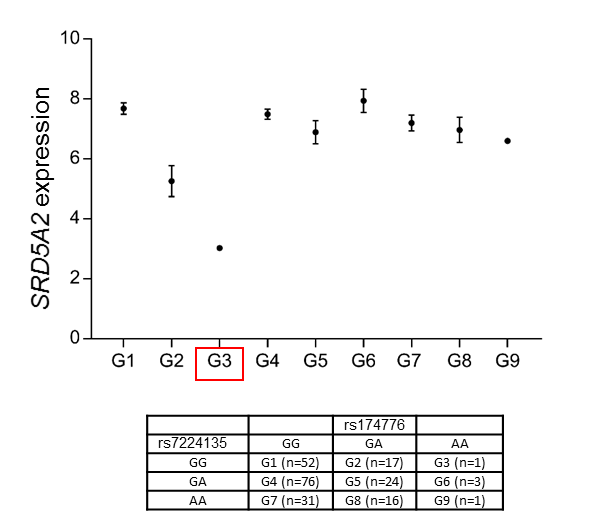

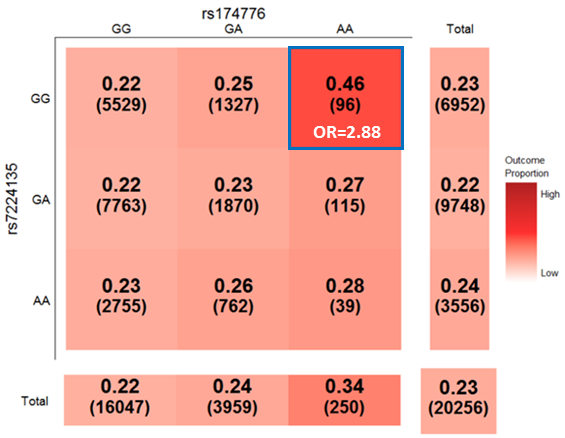


Note: mean± standard error, *SRD5A2* expression levels are lower in those with aggressive PCa than those with non-aggressive PCa (eQTL p=2.5x10^-9^).
